# Influence of the mean airway pressure trajectory on the mortality and AKI occurrence in septic shock patients with mechanical ventilation: insights from the MIMIC-IV database

**DOI:** 10.3389/fmed.2025.1552336

**Published:** 2025-03-05

**Authors:** Yukang Dong, Changyan Yang, Run Sun, Jiangquan Fu, Rui Huang, Jia Yuan, Ying Wang, Jinni Wang, Feng Shen

**Affiliations:** ^1^Department of Intensive Care Unit, Guizhou Medical University Affiliated Hospital, Guiyang, China; ^2^Department of Emergency, Guizhou Provincial People’s Hospital, Guiyang, China; ^3^Department of Emergency Intensive Care Unit, Guizhou Medical University Affiliated Hospital, Guiyang, China

**Keywords:** mean airway pressure, septic shock, acute kidney injury, mortality, MIMIC-IV database

## Abstract

**Background:**

Mean airway pressure (Pmean) is a known prognostic marker for mortality and adverse outcomes in mechanically ventilated patients. However, most previous studies have relied on static measurements, leaving the impact of Pmean trajectory on clinical outcomes in septic shock patients unclear. This study aimed to investigate the effect of Pmean trajectory on survival rates and acute kidney injury (AKI) incidence in septic shock patients undergoing mechanical ventilation (MV).

**Methods:**

A retrospective cohort study was implemented utilizing sepsis patient data from the MIMIC-IV database. Group-based trajectory modeling (GBTM) was applied to identify distinct Pmean trajectory groups among septic shock patients. Cox proportional hazards and logistic regression models were utilized to analyze associations between Pmean trajectory and both mortality and AKI incidence. A causal mediation analysis evaluated the intermediary effect of cumulative fluid balance over the first 72 h post-ICU admission.

**Results:**

A total of 956 eligible patients were included. Based on model fitting criteria, five distinct Pmean trajectory groups were identified: group 1 (low-stable), group 2 (high-descend), group 3 (medium-ascend), group 4 (high-stable), and group 5 (higher-stable). Compared to the low-stable trajectory (group 1), trajectories in groups 3, 4, and 5 were associated with significantly higher 30-day mortality risks (HR = 1.40, 95% CI = 1.03–1.88; HR = 1.47, 95% CI = 1.01–2.13; HR = 2.54, 95% CI = 1.53–4.2, respectively), while group 2 exhibited similar mortality rates to group 1 (HR = 0.88, 95% CI = 0.60–1.30). Logistic regression analyses revealed that groups 3, 4, and 5 were also significant risk factors for AKI occurrence (*p* < 0.05), with group 1 as the reference. Mediation analysis revealed that 20.5% (95% CI = 0.106–0.40) of the Pmean trajectory effect on AKI occurrence was mediated through cumulative fluid balance.

**Conclusion:**

Pmean trajectories were strongly associated with mortality and AKI incidence in septic shock patients receiving MV.

## 1 Introduction

Mechanical ventilation (MV) is a critical life-support measure for patients with acute respiratory distress syndrome (ARDS), with approximately 40% of ARDS cases linked to sepsis (1). The local and systemic inflammation caused by sepsis can worsen ventilator-induced lung injury (VILI) (2–4), making sepsis a frequent contributor to lung damage and increasing vulnerability to VILI during MV (5). Ventilation protocols for sepsis are typically adapted from evidence-based guidelines for ARDS, focusing on lung-protective strategies to prevent injury due to excessive pressure or volume on lung tissue. Two primary parameters in lung-protective ventilation—driving pressure (Pdriv) and mechanical power—have been shown to closely relate to mortality (6). However, in a large observational study, plateau pressure was recorded in only 40.1% of ARDS patients (7), with even fewer cases involving measurements of Pdriv and mechanical power that require plateau pressure. Despite adherence to small tidal volume strategies aimed at lung protection, severe cases of ARDS often experience rapid declines in circulation and perfusion, underscoring a limitation of these conventional approaches. For septic shock, ventilation strategies may need to go beyond lung protection alone to address the hemodynamic impact of respiratory mechanics, enhancing oxygen delivery and ultimately improving organ perfusion. This highlights the need for an optimal airway pressure indicator that simultaneously promotes both pulmonary and circulatory protection in septic shock patients undergoing invasive MV.

Mean airway pressure (Pmean) is a continuously monitored, dynamic parameter on mechanical ventilators, reflecting both phases of the respiratory cycle and indicating the mechanical power influenced by the ventilator mode (8). Pmean provides a more accurate measure of hemodynamic effects during MV than plateau pressure (Pplat) and Pdriv (9, 10). It represents the stress exerted on the respiratory system throughout ventilation and has been linked to 90-day mortality in patients with acute respiratory failure (11). However, prior studies have predominantly assessed static Pmean values, which may introduce bias and clinical confounding when dynamic variations over time are not considered. While Pmean is associated with lung-protective ventilation strategies, oxygenation, cardiovascular function, and the risk of VILI, its predictive value regarding outcomes in septic shock patients on MV remains unexplored. By focusing on long-term prognostic indicators instead of static measurements, it may be possible to identify patient heterogeneity and adjust management strategies proactively (12). This study probed into the relationship between dynamic Pmean trajectories and ICU clinical outcomes in patients receiving MV, providing a theoretical basis to enhance current treatment protocols.

## 2 Methods

### 2.1 Data source

This study utilized a cohort of septic shock patients from the Medical Information Mart for Intensive Care-IV (MIMIC-IV) (version 2.0), which includes clinical data from patients admitted to the Beth Israel Deaconess Medical Center between 2012 and 2019. Our research team obtained access to this data by completing the Protecting Human Research Participants test and the National Institutes of Health training (certification number: 44545022/39719844). The institutional review boards of Beth Israel Deaconess Medical Center and the Massachusetts Institute of Technology approved the creation of the MIMIC-IV database. Informed consent was waived, as all patient data were anonymized. This study adhered to the “Strengthening the Reporting of Observational Studies in Epidemiology” (STROBE) guidelines.

### 2.2 Study population

All patients recorded in the MIMIC-IV database were evaluated for inclusion. The eligibility criteria included: (1) ICU admission with a diagnosis of sepsis, as defined by the Sepsis 3.0 criteria, requiring a Sequential Organ Failure Assessment (SOFA) score of 2 or more in the context of suspected or confirmed infection; (2) blood lactate levels ≥2 mmol/L and administration of norepinephrine within the first 24 h after ICU admission; (3) initiation of invasive MV within 24 h of ICU entry; (4) continuous MV for a minimum of 72 h; (5) age ≥ 18 years; and (6) inclusion limited to the patient’s first ICU admission only (13). Exclusion criteria were: (1) Patients with chronic kidney disease (CKD); (2)pre-existing acute kidney injury (AKI) prior to ICU admission; and (3) lack of Pmean data at initiation or fewer than four Pmean measurements within the initial 72 h in the ICU.

### 2.3 Covariates

Data extraction was done utilizing Structured Query Language (SQL) and PostgreSQL (version 9.6). Demographic details gathered within the first 24 h of admission included age, gender, ethnicity (categorized as white, black, or other), body mass index (BMI), Simplified Acute Physiology Score II (SAPS II), Sequential Organ Failure Assessment (SOFA) score, and comorbidities such as cerebrovascular disease, congestive heart failure, chronic obstructive pulmonary disease, malignancy, and ARDS. Vital signs and laboratory values from the first ICU Day were averaged. Interventions administered within the first 24 h included neuromuscular blocking agents (NMBG) like cisatracurium, vecuronium bromide, or rocuronium, renal replacement therapy (RRT), prone position ventilation, and fluid balance. Daily ventilator variables were evaluated for continuity and included Pmean, tidal volume (Vt), positive end-expiratory pressure (PEEP), driving pressure, and mechanical power (MP), which were extracted as time-weighted averages (TWA) by dividing the area under the curve for each variable by time.

Mechanical power was estimated as follows:

MP (J/min) = 0.098 × respiratory rate × tidal volume × (Ppeak – 0.5 × dynamic ΔP) (14, 15)

where the dynamic driving pressure (ΔP) = Ppeak – PEEP (15, 16).

For the time-weighted average of Pmean (TWA-Pmean), we used the following equation: TWA-Pmean = ∑ (Pmean*ᵢ* × Δ*Tᵢ*) ÷ ∑ Δ*Tᵢ*.

Where: Pmean*ᵢ* represents the ith recorded Pmean measurement. Δ*Tᵢ* represents the time interval between the ith and the (*i* + 1)th Pmean measurement. TWA-MP, TWA-Pdriv, TWA-PEEP, and TWA-Tidal volume were calculated using the same method as TWA-Pmean. Pmean was replaced by mechanical power, driving pressure, peep and tidal volume.

### 2.4 Definitions and outcomes

The primary exposure assessed in this study was the Pmean trajectory. The main outcome of interest was 30-day mortality, while secondary outcomes included cumulative fluid balance within the first 72 h, ICU and hospital mortality, ICU and hospital length of stay, duration of MV, and incidence of AKI within 7 days of ICU admission. AKI was defined in the light of the Kidney Disease: Improving Global Outcomes (KDIGO) guidelines (17), as either a rise in serum creatinine to at least 1.5 times the baseline, an increase in serum creatinine by at least 0.3 mg/dL within 48 h, or urine output below 0.5 mL/(kg•h) for a minimum of 6 h. The baseline for serum creatinine was the first measurement taken at ICU admission (18).

### 2.5 Statistical analysis

Categorical variables were described utilizing frequencies and percentages, while continuous variables were summarized as medians with interquartile ranges (IQR) for skewed distributions or as means with standard deviations (SD) for normally distributed data. Baseline characteristics among the trajectory groups were compared using one-way analysis of variance (ANOVA) or Kruskal-Wallis tests for continuous variables, and chi-square tests for categorical variables.

Group-based trajectory modeling (GBTM) was adopted to identify distinct longitudinal patterns of Pmean. This involved a two-stage iterative model-fitting process: initially, polynomial functions were applied to trajectories to determine the number of groups, with models ranging from one (indicating no significant trajectory) to six groups. After selecting the optimal number of groups, the model identified the shape of each trajectory. Model fit was assessed according to several criteria (19): (1) Bayesian Information Criterion (BIC), with values closer to 0 reflecting a better fit; (2) average post-test grouping probability (Avepp), with values above 0.7 signify reliable subgroup classification; (3) Bayesian factor logarithm (2log_e_ [B10]), approximately twice the difference in BIC values (2 ΔBIC) between models, where values >6 indicate significant model differentiation and values <2 favor a more concise model; (4) requirement that each trajectory group include at least 5% of the population; and (5) consistency between estimated group probability and the assigned population proportion. Model simplicity and clinical interpretability were also considered.

Kaplan–Meier curves were generated to compare survival across Pmean trajectory groups. Associations between trajectory groups or individual Pmean levels at specific time points and study outcomes (mortality and AKI incidence) were analyzed utilizing univariate and multivariable forward stepwise Cox regression or logistic regression models, with entry criteria for variables set at *p* < 0.1 and removal criteria at *p* < 0.05. To reduce overfitting from multicollinearity, variance inflation factors (VIF) were calculated, excluding variables with VIF ≥ 5. Restricted cubic splines were employed to model and visualize the association between Pmean during distinct intervals (first 24 h, second 24 h, and third 24 h) and subsequent 30-day mortality.

Causal mediation analysis (CMA) was conducted to examine whether cumulative fluid balance, as a potential factor influencing mortality and AKI incidence (20), could mediate the effects of mean airway pressure trajectories on these outcomes. The total effect, average direct effect (ADE), and average causal mediation effect (ACME) were calculated. Subgroup analyses were conducted based on age, gender, BMI, prone position ventilation, use of NMBAS, SAPS II, SOFA score, and comorbidities. Interaction terms between trajectory group and stratification variables were included to assess potential interactions.

Missing values were addressed utilizing chained equations within a multivariate imputation framework, assuming values were missing at random. The percentage of missing data is provided in Supplementary Table S1.

All analyses were done utilizing R version 4.1.2 (R Foundation), Free Statistics version 1.9, and Stata version 17 (StataCorp LLC, College Station, TX, United States), with statistical significance established at *p* < 0.05.

## 3 Results

### 3.1 Pmean trajectories, baseline characteristics, and clinical outcomes

**Figure 1:**
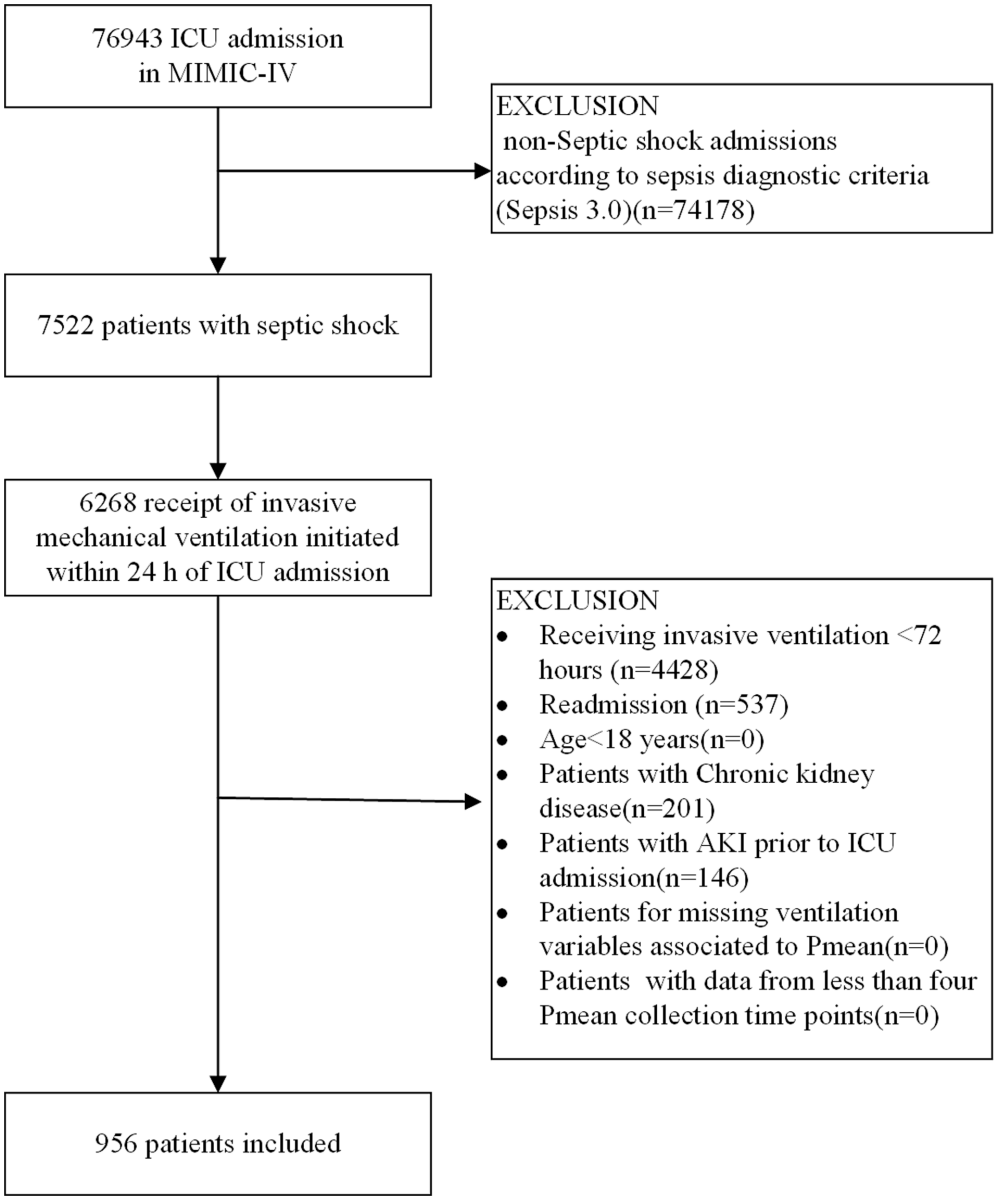
Flowchart of patient inclusion procedure. ICU, intensive care unit; Pmean, mean airway pressure.

**Figure 2:**
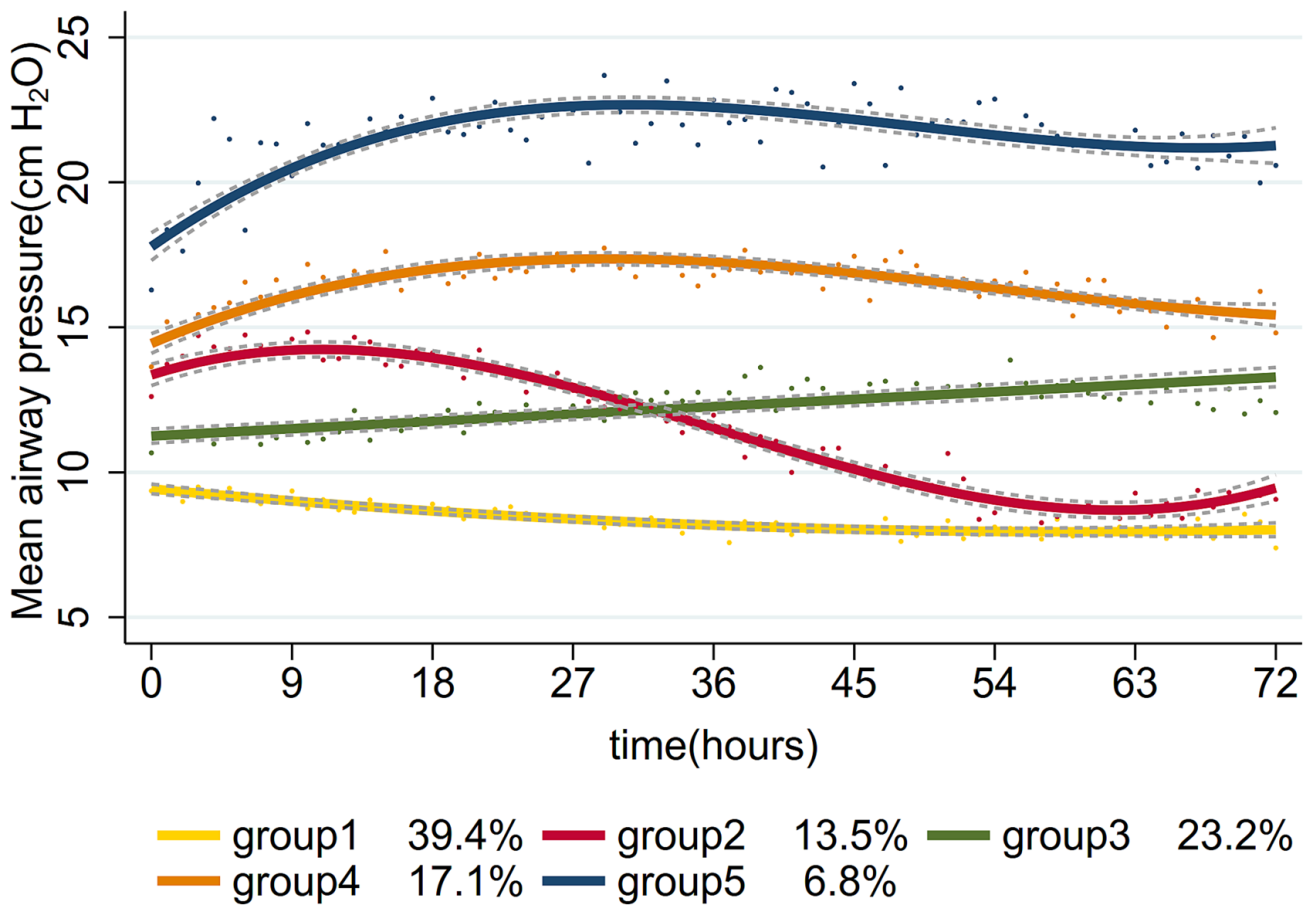
Mean airway pressure-based trajectories of patients with septic shock with mechanical ventilation.

The study process is outlined in the flowchart presented in Figure 1. By referring the inclusion and exclusion criteria, 956 patients were enrolled. We identified optimal Pmean trajectories by evaluating the fitting parameters described in the statistical analysis section. The posterior probabilities and goodness-of-fit metrics for GBTM across various group numbers (two to six) and trajectory shapes (linear, quadratic, and cubic) are detailed in Supplementary Tables S2, S3. After considering clinical relevance and interpretability, a five-group trajectory model was selected. This model yielded five linear trajectories (Figure 2). Based on Pmean characteristics across these trajectories, the groups were classified as follows: Group 1 (low-stable), comprising 377 patients (39.4%) with stable, low Pmean levels; Group 2 (high-descend), with 129 patients (13.5%) whose Pmean gradually decreased over time; Group 3 (medium-ascend), including 222 patients (23.2%) with progressively increasing Pmean; Group 4 (high-stable), comprising 163 patients (17.1%) with consistently high Pmean levels; and Group 5 (higher-stable), including 65 patients (6.8%) who maintained very high Pmean levels without fluctuation.

The baseline characteristics are detailed in Table 1. The mean age of participants was 60.3 years, with male patients representing 58.4% and white patients comprising approximately 52.9% of the cohort. Alarming rates of 30-day mortality and AKI incidence within 7 days were observed at 39 and 41%, respectively. Notably, patients in Group 5 had a younger demographic profile but presented with more severe conditions, including significantly elevated cumulative fluid balance over 72 h, extended ICU stays, longer durations of MV, and elevated mortality and AKI rates. Specifically, Group 5 showed a 30-day mortality rate of 50.8%, ICU mortality of 43.1%, hospital mortality of 46.2%, and an AKI incidence of 98.5% within 7 days. Table 1 provides detailed time-weighted mean airway pressure values for each time interval across the five groups. Significant differences in daily Pmean variations were observed across all time points among these groups, as shown in Table 1 and Supplementary Figure S1.

**Table 1:** Baseline characteristics and clinical outcomes among five trajectory groups.

Characteristics	Total	Group 1	Group 2	Group 3	Group 4	Group 5	*p*_value
	(*n* = 956)	(*n* = 377)	(*n* = 129)	(*n* = 222)	(*n* = 163)	(*n* = 65)	
Age (years)	60.3 ± 17.3	63.7 ± 17.3	61.2 ± 17.4	59.4 ± 16.7	57.2 ± 16.0	49.5 ± 16.0	<0.001
Gender (male)	558 (58.4)	215 (57)	73 (56.6)	129 (58.1)	99 (60.7)	42 (64.6)	0.759
BMI (kg/m^2^)	29.3 ± 7.9	26.9 ± 6.5	29.3 ± 7.6	29.2 ± 6.9	31.8 ± 8.6	36.6 ± 10.5	<0.001
Ethnicity, *n* (%)							0.022
White	506 (52.9)	218 (57.8)	72 (55.8)	114 (51.4)	79 (48.5)	23 (35.4)	
Black	63 (6.6)	22 (5.8)	13 (10.1)	14 (6.3)	9 (5.5)	5 (7.7)	
Other	387 (40.5)	137 (36.3)	44 (34.1)	94 (42.3)	75 (46)	37 (56.9)	
SAPS II score	49.9 ± 15.1	47.8 ± 14.9	51.9 ± 15.3	49.8 ± 15.3	51.9 ± 15.3	52.8 ± 13.7	0.006
SOFA score	11.3 ± 3.8	9.8 ± 3.7	11.7 ± 3.5	11.5 ± 3.8	12.8 ± 3.1	13.7 ± 3.3	<0.001
CHF, *n* (%)	206 (21.5)	75 (19.9)	39 (30.2)	44 (19.8)	35 (21.5)	13 (20)	0.144
CAD, *n* (%)	192 (20.1)	68 (18)	30 (23.3)	49 (22.1)	36 (22.1)	9 (13.8)	0.364
COPD, *n* (%)	121 (12.7)	33 (8.8)	22 (17.1)	28 (12.6)	35 (21.5)	3 (4.6)	<0.001
ARDS, *n* (%)	240 (25.1)	59 (15.6)	33 (25.6)	56 (25.2)	61 (37.4)	31 (47.7)	<0.001
Malignancy, *n* (%)	149 (15.6)	68 (18)	20 (15.5)	32 (14.4)	26 (16)	3 (4.6)	0.095
NMBA use, *n* (%)	98 (10.3)	16 (4.2)	12 (9.3)	34 (15.3)	24 (14.7)	12 (18.5)	<0.001
RRT use, *n* (%)	59 (6.2)	9 (2.4)	9 (7)	15 (6.8)	15 (9.2)	11 (16.9)	<0.001
Prone position ventilation, *n* (%)	71 (7.4)	25 (6.6)	10 (7.8)	18 (8.1)	10 (6.1)	8 (12.3)	0.5
Fluid balance (L)	7.5 ± 5.8	6.4 ± 5.3	7.0 ± 5.3	7.7 ± 5.9	9.1 ± 6.5	9.6 ± 6.4	<0.001
Heart rate (bpm)	93.8 ± 18.3	91.2 ± 17.7	93.9 ± 16.7	92.5 ± 18.9	97.7 ± 18.7	102.6 ± 18.1	<0.001
MAP (mmHg)	75.6 ± 8.5	75.6 ± 8.2	75.8 ± 8.4	75.1 ± 8.7	75.5 ± 8.6	76.6 ± 10.3	0.806
Respiratory rate (bpm)	21.4 ± 4.3	19.2 ± 3.4	22.6 ± 3.7	21.2 ± 4.1	23.9 ± 4.2	26.1 ± 3.9	<0.001
SpO_2_, %	93 (89, 95)	95 (92, 97)	92 (88, 94)	92(90, 95)	90(86 93)	87(75, 89)	<0.001
PF ratio	248.1 ± 108.7	307.7 ± 113.3	237.2 ± 81.6	232.4 ± 91.5	179.3 ± 60.6	156.4 ± 84.6	<0.001
Hemoglobin (g/dl)	11.2 ± 2.6	10.9 ± 2.4	11.3 ± 2.8	10.9 ± 2.6	11.6 ± 2.7	12.1 ± 3.0	<0.001
Platelet (k/uL)	195.1 ± 113.1	197.7 ± 114.9	212.7 ± 120.7	187.2 ± 116.2	179.4 ± 102.8	211.1 ± 96.1	0.065
PH	7.3 ± 0.1	7.3 ± 0.1	7.3 ± 0.1	7.3 ± 0.1	7.2 ± 0.1	7.2 ± 0.1	<0.001
PaCO_2_ (mmHg)	44.1 ± 14.3	40.7 ± 9.8	45.8 ± 15.6	42.9 ± 13.7	49.2 ± 18.3	51.1 ± 17.6	<0.001
Chloride (mEq/L)	106.1 ± 7.1	106.4 ± 7.0	107.1 ± 7.7	105.8 ± 6.5	105.6 ± 7.7	105.0 ± 7.2	0.229
Sodium (mEq/L)	138.2 ± 6.0	138.3 ± 6.1	139.3 ± 6.6	137.3 ± 5.4	137.9 ± 6.2	138.6 ± 5.3	0.043
Bicarbonate (mEq/L)	19.4 ± 5.0	19.6 ± 4.4	19.8 ± 5.4	19.1 ± 5.3	19.2 ± 5.3	19.0 ± 5.5	0.592
Lactate (mmol/L)	3.6 (2.6, 5.3)	3.3 (2.6, 4.8)	3.4 (2.5, 4.8)	3.7 (2.6, 6.0)	4.1 (2.7, 5.6)	4.0 (2.9, 5.3)	0.008
Neutrophil (k/uL)	76.1 ± 17.8	77.4 ± 17.6	78.2 ± 15.8	75.5 ± 18.1	72.9 ± 19.5	77.1 ± 15.5	0.252
Creatinine (g/dL)	1.4 ± 1.0	1.2 ± 0.7	1.4 ± 1.4	1.3 ± 1.0	1.5 ± 1.0	1.6 ± 0.9	0.009
TWA-MP (J/min)	17.5 ± 7.9	12.3 ± 4.5	20.0 ± 5.2	16.5 ± 5.5	23.9 ± 7.0	30.8 ± 8.5	<0.001
TWA-Pdriv (cm H_2_O)	12.3 ± 3.8	11.2 ± 3.2	12.2 ± 4.5	12.8 ± 3.7	13.0 ± 4.0	14.1 ± 3.6	<0.001
TWA-Tidal volume (ml)	606.6 ± 443.1	615.1 ± 291.2	560.6 ± 145.0	640.4 ± 709.2	592.2 ± 464.6	570.0 ± 308.7	0.488
TWA-PEEP (cm H_2_O)	9.0 (6.0, 12.0)	5.5 (5.0, 8.0)	11.0 (10.0, 12.0)	9.0 (7.0, 10.0)	14.0 (12.0, 15.4)	19.0 (16.0, 21.0)	<0.001
TWA-Pmean^a^ (cm H_2_O)	12.4 ± 4.2	8.9 ± 1.5	14.3 ± 2.2	11.6 ± 1.7	16.3 ± 2.5	21.2 ± 3.7	<0.001
TWA-Pmean^b^ (cm H_2_O)	12.1 ± 4.6	8.2 ± 1.4	11.7 ± 2.2	12.5 ± 1.8	17.0 ± 2.1	22.2 ± 2.9	<0.001
TWA-Pmean^c^ (cm H_2_O)	11.5 ± 4.7	7.8 ± 1.7	9.1 ± 2.0	12.6 ± 2.1	16.1 ± 2.8	21.8 ± 3.7	<0.001
Clinical outcomes							
Cumulative fluid balance_72h_ (L)	11.32 ± 9.6	9.1 ± 8.0	10.2 ± 8.8	11.8 ± 9.2	14.2 ± 11.1	17.8 ± 12.2	<0.001
Duration of ventilation (days)	8.2 ± 7.3	6.7 ± 6.6	7.8 ± 6.6	8.8 ± 6.5	9.8 ± 7.3	11.0 ± 12.3	<0.001
Length of ICU stay (days)	10.8 ± 8.6	9.4 ± 7.4	10.7 ± 8.3	11.4 ± 7.9	12.4 ± 8.8	13.4 ± 14.1	<0.001
Length of hospital stay (days)	18.0 ± 15.0	18.5 ± 15.0	18.5 ± 16.1	17.5 ± 15.7	17.1 ± 12.3	17.6 ± 17.3	0.849
ICU mortality, *n* (%)	264 (27.6)	80 (21.2)	24 (18.6)	73 (32.9)	59 (36.2)	28 (43.1)	<0.001
hospital mortality, *n* (%)	319 (33.4)	106 (28.1)	35 (27.1)	84 (37.8)	64 (39.3)	30 (46.2)	0.003
30-day mortality, *n* (%)	345 (36.1)	120 (31.8)	38 (29.5)	88 (39.6)	66 (40.5)	33 (50.8)	0.007
AKI, *n* (%)	880 (92.1)	327 (86.7)	117 (90.7)	214 (96.4)	158 (96.9)	64 (98.5)	<0.001
AKI stage							0.718
AKI I, *n* (%)	718 (81.6)	270 (82.6)	95 (81.2)	177 (82.7)	125 (79.1)	51 (79.7)	
AKI II, *n* (%)	155 (17.6)	56 (17.1)	21 (17.9)	36 (16.8)	30 (19)	12 (18.8)	
AKI III, *n* (%)	7 (0.8)	1 (0.3)	1 (0.9)	1 (0.5)	3 (1.9)	1 (1.6)	

For skewed variables, the data were presented as mean ± SD or median (IQR), and for categorical variables, as numbers (proportions).

BMI, body mass index; SAPS II, simplified acute physiology score II; SOFA, sequential organ failure assessment; NMBA, neuromuscular blocker agent; RRT, renal replacement therapy; CHF, congestive heart failure; CAD, cerebrovascular disease; ARDS, acute respiratory distress syndrome; COPD, chronic obstructive pulmonary disease; bpm, beats per minute; MAP, mean arterial blood pressure; SpO_2_, pulse oximetry; PaCO2, arterial carbon dioxide tension; PaO_2_, arterial oxygen tension; PEEP, positive end-expiratory pressure; PF ratio, PaO_2_/FiO_2_ ratio; Pdriv, driving pressure; MP, mechanical power, TWA, time-weighted averages; AKI, acute kidney injury.

a First 24-h.

b Second 24-h.

c Third 24-h.

### 3.2 TWA-Pmean levels at various time points and 30-day mortality

**Figure 3:**
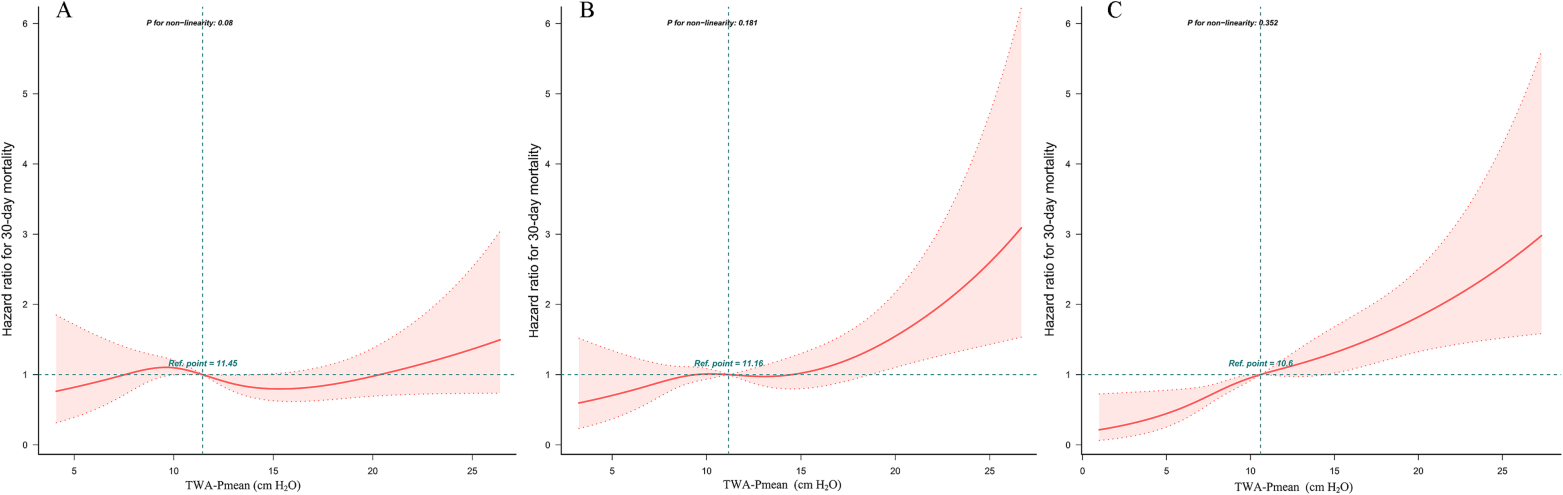
Restricted cubic spline (RCS) model showing the association between TWA-Mean airway pressure levels and 30-day mortality in patients with septic shock. **(A)** The first 24-h; **(B)** The second 24-h; **(C)** The third 24-h.

The relationship between TWA-Pmean levels at different time points within the first 72 h following ICU admission and 30-day mortality is detailed in Supplementary Table S4. Elevated TWA-Pmean levels during the second and third 24-h periods post-ICU admission were associated with an increased risk of 30-day mortality in patients with septic shock (hazard ratio [HR] = 1.04, 95% confidence interval [CI]: 1.01–1.06; HR = 1.06, 95% CI: 1.03–1.08, respectively), while no significant association was observed between TWA-Pmean levels during the first 24 h and 30-day mortality (*p* = 0.11). The multivariable Cox proportional hazards model indicated that elevated TWA-Pmean levels during the second and third 24-h intervals (HR = 1.05, 95% CI: 1.01–1.08; HR = 1.09, 95% CI: 1.06–1.12, respectively) were linked to a higher risk of 30-day mortality among patients with septic shock. Figure 3 and Supplementary Table S4 illustrate the association between TWA-Pmean levels at different time points and 30-day mortality.

### 3.3 Association between Pmean trajectories and mortality

**Figure 4:**
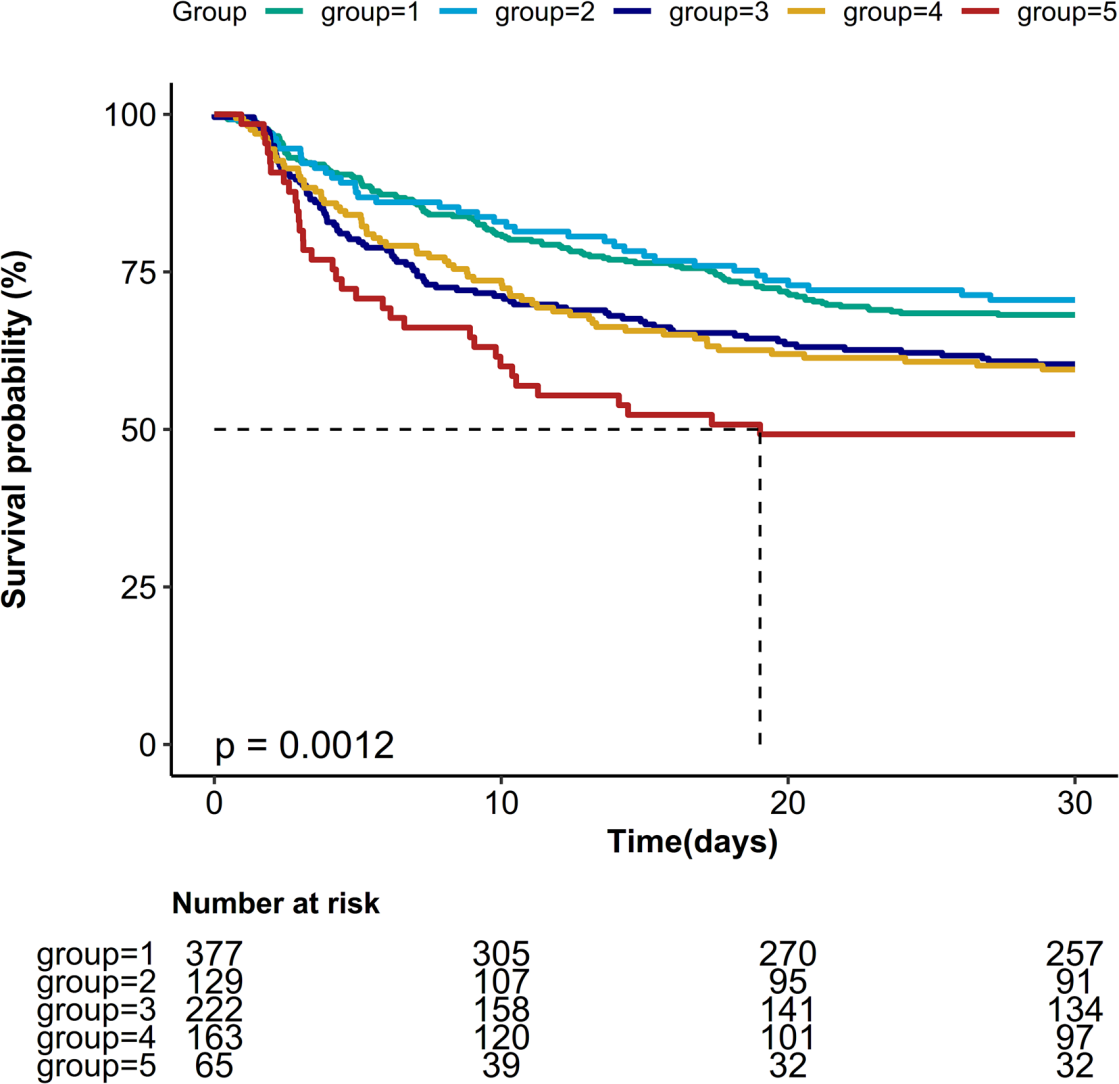
Kaplan–Meier survival estimates of 30-day mortality among each mean airway pressure trajectory.

Kaplan–Meier survival curves revealed significant differences in 30-day mortality rates across the five Pmean trajectory groups (Figure 4, *p* = 0.0012). The association between various Pmean trajectories and 30-day mortality is illustrated in Table 2. Compared to the low-stable trajectory group, the medium-ascend, high-stable, and higher-stable trajectory groups shared correlation with a higher risk of 30-day mortality (HR = 1.37, 95% CI: 1.04–1.8; HR = 1.38, 95% CI: 1.03–1.87; HR = 1.98, 95% CI: 1.35–2.91, respectively), while no significant association was found for the high-descend trajectory group. After adjusting for potential confounders—including age, gender, BMI, SAPS II score, SOFA score, fluid balance, respiratory rate, SpO_2_ levels, PF ratio, pH, bicarbonate concentration, lactate, and sodium—the negative impact of the medium-ascend, high-stable, and higher-stable trajectory groups on 30-day survival remained statistically significant (HR = 1.40, 95% CI: 1.03–1.88; HR = 1.47, 95% CI: 1.01–2.13; HR = 2.54, 95% CI: 1.53–4.2, respectively). Similar findings were observed in multivariable logistic regression analyses for ICU and hospital mortality outcomes (Table 2).

**Table 2:** Hazard ratio or odds ratio for risks of mortality by different Pmean trajectory patterns.

Variable	N. total	Crude		Model 1		Model 2		Model 3	
		HR/OR (95% CI)	*p*_value	HR/OR (95% CI)	*p*_value	HR/OR (95% CI)	*p*_value	HR/OR (95% CI)	*p*_value
30-day mortality									
Group 1	377	Reference		Reference		Reference		Reference	
Group 2	129	0.92 (0.64 ~ 1.32)	0.644	0.97 (0.67 ~ 1.4)	0.86	0.85 (0.58 ~ 1.25)	0.415	0.88 (0.6 ~ 1.3)	0.517
Group 3	222	1.37 (1.04 ~ 1.8)	0.027	1.48 (1.12 ~ 1.96)	0.006	1.39 (1.03 ~ 1.88)	0.031	1.4 (1.03 ~ 1.88)	0.03
Group 4	163	1.38 (1.03 ~ 1.87)	0.034	1.63 (1.19 ~ 2.22)	0.002	1.45 (1.01 ~ 2.09)	0.046	1.47 (1.01 ~ 2.13)	0.043
Group 5	65	1.98 (1.35 ~ 2.91)	0.001	2.76 (1.82 ~ 4.2)	<0.001	2.29 (1.39 ~ 3.78)	0.001	2.54 (1.53 ~ 4.2)	<0.001
ICU mortality									
Group 1	377	Reference		Reference		Reference		Reference	
Group 2	129	0.85 (0.51 ~ 1.41)	0.526	0.9 (0.54 ~ 1.51)	0.701	0.72 (0.41 ~ 1.24)	0.236	0.74 (0.42 ~ 1.31)	0.303
Group 3	222	1.82 (1.25 ~ 2.64)	0.002	2.03 (1.38 ~ 2.97)	<0.001	1.88 (1.23 ~ 2.86)	0.003	1.87 (1.22 ~ 2.88)	0.004
Group 4	163	2.11 (1.41 ~ 3.15)	<0.001	2.52 (1.65 ~ 3.86)	<0.001	2.12 (1.27 ~ 3.55)	0.004	2.18 (1.28 ~ 3.71)	0.004
Group 5	65	2.81 (1.62 ~ 4.87)	<0.001	3.99 (2.2 ~ 7.26)	<0.001	2.86 (1.38 ~ 5.94)	0.005	3.14 (1.48 ~ 6.69)	0.003
Hospital mortality									
Group 1	377	Reference		Reference		Reference		Reference	
Group 2	129	0.95 (0.61 ~ 1.49)	0.829	1.03 (0.65 ~ 1.62)	0.904	0.82 (0.5 ~ 1.35)	0.441	0.85 (0.51 ~ 1.4)	0.514
Group 3	222	1.56 (1.09 ~ 2.21)	0.014	1.77 (1.23 ~ 2.55)	0.002	1.65 (1.1 ~ 2.47)	0.015	1.64 (1.09 ~ 2.46)	0.018
Group 4	163	1.65 (1.12 ~ 2.43)	0.011	2.04 (1.36 ~ 3.07)	0.001	1.73 (1.05 ~ 2.85)	0.03	1.76 (1.05 ~ 2.93)	0.031
Group 5	65	2.19 (1.28 ~ 3.75)	0.004	3.34 (1.86 ~ 5.99)	<0.001	2.46 (1.2 ~ 5.03)	0.014	2.61 (1.25 ~ 5.44)	0.01

Model 1: adjusted for age, gender, and BMI.

Model 2: adjusted for age, gender, BMI, SAPS II score, SOFA score, fluid balance respiratory rate, SpO_2_ and PF ratio.

Model 3: adjusted for age, gender, BMI, SAPS II score, SOFA score, fluid balance, respiratory rate, SpO_2_, PF ratio, PH, Bicarbonate, Lactate and sodium.

HR, hazard ratio; OR, odds ratio; CI, confidence interval; PF ratio, PaO_2_/FiO_2_ ratio; SpO_2_, pulse oximetry.

### 3.4 Association between Pmean trajectories and AKI occurrence

**Figure 5:**
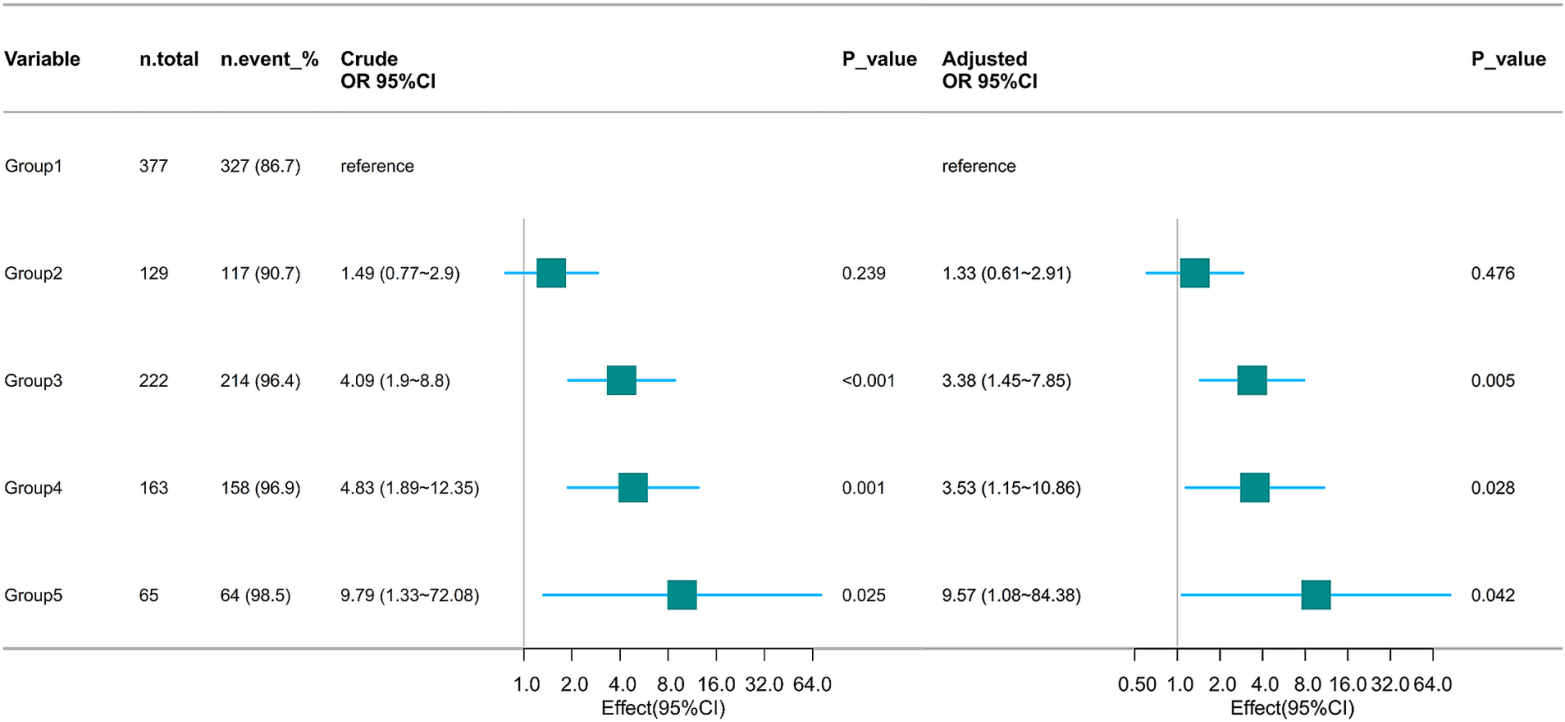
Association between mean airway pressure trajectory and AKI occurrence. OR, Odds Ratio; CI, confidence interval.

Multivariate logistic regression analysis, adjusted for age, gender, BMI, SOFA score, SAPS II score, mean arterial pressure (MAP), heart rate, respiratory rate, PaO_2_/FiO_2_ ratio, fluid balance, chloride levels, sodium concentration, lactate levels, pH, and hemoglobin concentration, showed that relative to the low-stable group, patients in the medium-ascend trajectory group (Odds Ratio [OR] = 3.38, 95% CI: 1.45–7.85), high-stable trajectory group (OR = 3.53, 95% CI: 1.15–10.86), and higher-stable trajectory group (OR = 9.57, 95% CI: 1.08–84.38) had an increased risk of AKI occurrence. No significant association with AKI was noted for patients in the high-descend trajectory group (*p* = 0.476) (Figure 5).

### 3.5 CMA

**Figure 6:**
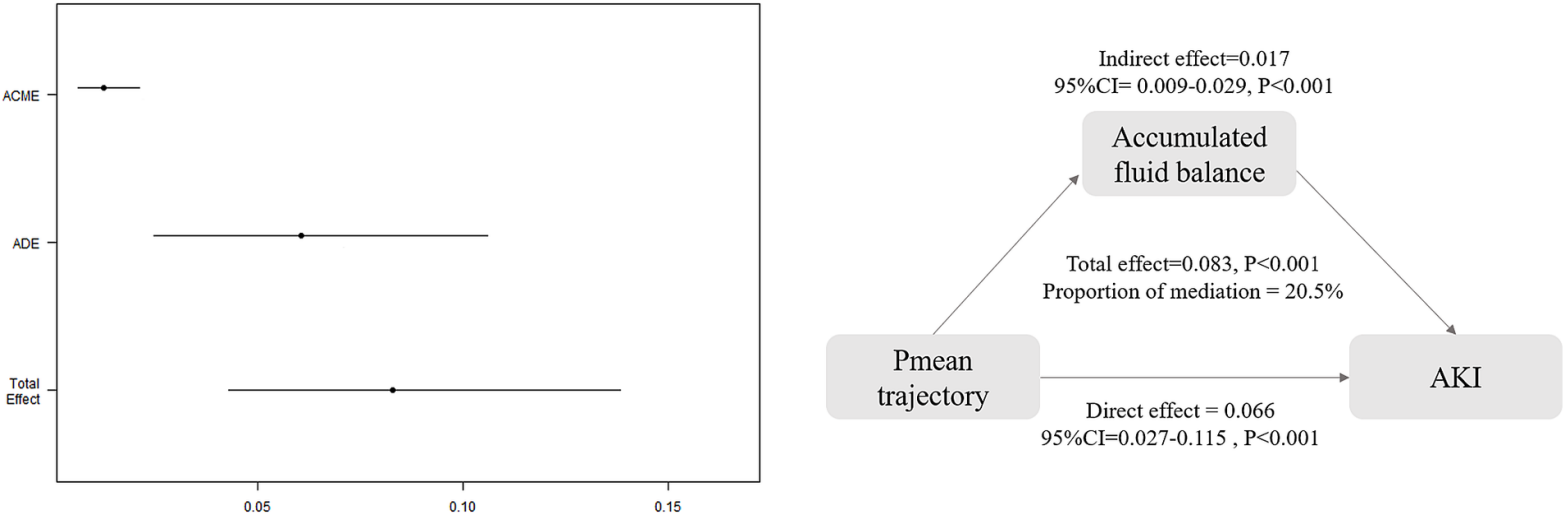
Causal mediation analysis for accumulated fluid balance. ACME, average causal mediation effect; ADE, average direct effect.

Mediation analysis indicated that 20.5% (95% CI: 0.106–0.40) of the association between Pmean trajectories and AKI risk was mediated by cumulative fluid balance over a 72-h period. The total effect was estimated at 0.083 (95% CI: 0.043–0.140; *p* < 0.001), with an ADE of 0.066 (95% CI: 0.027–0.115; *p* < 0.001) and an ACME of 0.017 (95% CI: 0.009–0.029; *p* < 0.001), as illustrated in Figure 6 and Supplementary Table S5. However, cumulative fluid balance as a mediator did not significantly affect the indirect association of Pmean trajectories with mortality outcomes, including 30-day, ICU, and hospital mortality (Supplementary Table S5).

### 3.6 Subgroup analysis

The results of the subgroup analysis are detailed in Supplementary Table S6. No statistical significance was observed in the interaction terms between trajectory class and any stratified variable, indicating a lack of interaction. A consistent correlation was found between Pmean classification and 30-day mortality risk in septic shock patients with varying baseline characteristics.

### 3.7 Sensitivity analysis

Since ventilator parameters are often dynamically adjusted within the first 24 h, which may influence the trajectory patterns identified by GBTM, we conducted a sensitivity analysis by applying GBTM starting from the second 24-h period. Similar Pmean trajectories were identified (Supplementary Figure S2). The distribution of patients across the low-stable, high-descend, medium-ascend, high-stable, and higher-stable Pmean trajectory groups was 408 (42.7%), 150 (15.7%), 205 (21.4%), 137 (14.3%), and 56 (5.9%), respectively. Compared to the low-stable trajectory group, patients in the moderate-increasing, high-stable, and higher-stable trajectory groups were associated with higher 30-day mortality, ICU mortality, and in-hospital mortality (Supplementary Table S7, Supplementary Figure S3). Furthermore, we also reconstructed Pmean trajectory patterns that closely resembled the original trajectories after excluding 59 patients who received RRT with possible effects on AKI (Supplementary Figure S5). In both sensitivity analyses, we observed that patients with medium-ascend, high-stable, or higher-stable Pmean trajectories had an increased risk of AKI compared to those with low-stable Pmean trajectories (Supplementary Figures S4, S6). However, the relationship between the higher-stable trajectory group and AKI incidence did not reach statistical significance, likely due to the small sample size and wide confidence intervals. Nonetheless, the direction of the effect remained consistent with the primary analysis, suggesting that these findings should be interpreted with caution.

## 4 Discussion

In this study, we identified five distinct Pmean trajectory patterns in septic shock patients using GBTM and found significant associations between these trajectories and mortality. Additionally, AKI occurrence was linked to these trajectory characteristics in mechanically ventilated septic shock patients. To our knowledge, this is the first study to elucidate the impact of longitudinal mean airway pressure on prognosis and AKI risk in this patient population.

The cornerstone of septic shock management lies in restoring tissue perfusion and maintaining hemodynamic stability. Septic shock often renders the lungs particularly vulnerable, necessitating MV for effective treatment. In such cases, VILI is a primary concern. Moreover, septic shock is often accompanied by septic cardiomyopathy, which further exacerbates circulatory instability (21). The broader concept of “cardio-protective” or “circulation-protective” ventilation (22, 23) has also emerged, emphasizing adjustments in ventilator settings to minimize the hemodynamic impact of positive pressure.

Pmean represents the mean alveolar pressure throughout the respiratory cycle (24), providing a more comprehensive measure of lung disease severity, respiratory compliance, and the extent of respiratory support needed, compared to plateau or driving pressures alone. Studies investigating outcomes in respiratory failure across both adult and pediatric populations have highlighted the importance of Pmean as a critical component of the oxygenation index (25, 26). Furthermore, Pmean is integral in calculating mechanical power; elevated Pmean indicates increased mechanical energy (14). A recent multicenter study found that Pmean was independently associated with 90-day mortality among ARDS patients undergoing MV, with predictive power similar to plateau or driving pressures (11). In this setting, Pmean may be particularly relevant in scenarios where spontaneous breathing efforts are excessive, or the respiratory drive is elevated (8).

As a key parameter affecting patient hemodynamics during MV, increased Pmean during high-frequency oscillations can lead to decreased cardiac output, which is directly associated with high mortality rates (27–30). Conversely, reducing Pmean during procedures such as cardiopulmonary resuscitation or myocardial revascularization surgery can mitigate negative impacts on hemodynamic parameters, including cardiac index, cardiac output, stroke volume, and MAP (23, 31). Su et al. documented previously that elevated Pmean and central venous pressure (CVP) were both linked to increased 28-day mortality (10). Additionally, Pan et al. showed that using SpO_2_ = 100%, PaCO_2_ ≥ 40 mmHg, and Pmean ≤ 10 cmH_2_O as ICU quality control targets improved 90-day survival, shortened the duration of MV, and reduced CVP (32). Thus, Pmean reflects both circulatory and pulmonary status during MV (33).

It is important to note that previous studies have often relied on single, static Pmean values, which may not accurately capture daily fluctuations in Pmean. Clinically, Pmean can be significantly affected by various ventilator settings. This limitation is particularly relevant in mechanically ventilated septic shock patients, whose physiological conditions are dynamic and rapidly evolving; thus, relying on static Pmean alone fails to fully represent the complexity of their oxygenation and hemodynamic profiles. In this study, we examined TWA Pmean values at various time points within the first 72 h of ICU admission. Notably, TWA-Pmean during the second and third 24-h intervals was independently and linearly associated with an increased risk of 30-day mortality, highlighting the importance of monitoring beyond the initial 24 h post-admission. Dynamic changes in relevant parameters should be closely observed. Additionally, time-weighted values may not fully capture the effects of prolonged versus transient elevations in Pmean on patient outcomes.

Based on Pmean changes during the initial 72 h following ICU admission, we categorized septic shock patients into five distinct trajectory groups. Our results indicated that patients in the medium-ascend, high-stable, and higher-stable trajectories had an increased risk of mortality compared to those in the low-stable trajectory; however, no significant association was noted between the high-descend trajectory and mortality. In the medium-ascend, high-stable, and higher-stable trajectories, Pmean consistently exceeded 10 cmH_2_O, supporting previous findings that adverse outcomes increase when Pmean surpasses this threshold (10, 32). At ICU admission, patients in the high-descend trajectory had higher initial Pmean values than those in the medium-ascend trajectory, but over 72 h, Pmean showed a downward trend, eventually falling below that of the medium-ascend group. This suggests that transient Pmean elevations may impact outcomes differently than either gradual increases or sustained elevations. This finding underscores that the relationship between initial Pmean and patient outcomes is not simply a matter of exceeding a threshold; rather, it depends on dynamic fluctuations and variability within Pmean trajectories, factors that may significantly influence prognosis. Longitudinal monitoring of Pmean may allow clinicians to develop more precise ventilation management strategies. Future research should explore the impact of extended Pmean trajectories on outcomes in sepsis patients.

Previous studies have established MV as a risk factor for AKI in critically ill patients (34, 35); however, the specific respiratory parameters contributing to AKI remain unclear. Our analysis of various Pmean trajectories revealed that the medium-ascend, high-stable, and higher-stable trajectories were linked to an increased risk of AKI relative to the low-stable trajectory. Prior research has shown that elevated CVP is closely linked to AKI development (36–38), and higher Pmean values have also been associated with elevated CVP (8). AKI occurrence due to elevated Pmean may be exacerbated by increased intrathoracic pressure, which raises right atrial pressure (CVP), thereby reducing the venous return gradient (39). In critically ill patients, reduced renal perfusion pressure—calculated as the difference between MAP and renal venous pressure—emerges as a contributing factor to AKI risk. Increased right atrial pressure propagates to increased renal venous pressure, lowering renal perfusion pressure and resulting in renal venous stasis (40, 41). This reduction in renal perfusion and increased venous stasis can adversely impact renal function, ultimately contributing to AKI (42). Additionally, we observed that higher cumulative fluid volume was associated with a higher Pmean trajectory. This may be explained by the fact that, in mechanically ventilated patients, positive airway pressure plays a significant role in promoting fluid retention. Increased airway pressure elevates intrathoracic pressure, which reduces central arterial blood volume. This reduction activates baroreceptors, leading to heightened vasomotor tone and increased reabsorption of sodium and water to restore blood volume (33, 43). The increased fluid balance could contribute to renal venous congestion and interstitial edema, both of which are recognized as independent risk factors for AKI (20). Elevated intrathoracic pressures induced by increased Pmean may further promote fluid retention (44). Using CMA, we demonstrated that the effect of Pmean trajectories on AKI incidence was partially mediated by cumulative fluid balance. It is thus reasonable to speculate that variations in Pmean trajectory may influence AKI onset during septic shock through their effects on cumulative fluid management. Although our findings partially elucidate the causal relationship between Pmean trajectories, fluid management practices, and short-term AKI incidence in septic shock patients, further validation in high-quality prospective studies is warranted.

This study has several limitations. First, the MIMIC database is a single-center source, and our investigation was conducted as a retrospective observational study. Due to potential bias and the lack of external validation from other databases, our findings may not be fully generalizable. Second, our research focused on a narrowly defined cohort of patients with septic shock, which limits the applicability of our results to the broader population of mechanically ventilated patients. Third, mean airway pressure is affected by multiple factors, none of which could be comprehensively assessed in this study. Fourth, the regression analysis did not fully adjust for baseline characteristics among patients in different trajectory groups, which may introduce inherent bias that could persist alongside the adverse effects of harmful exposures. Fifth, our study was limited by the lack of detailed hemodynamic data (e.g., cardiac output or other advanced monitoring parameters) and the absence of standardized information on specific mechanical ventilation modes within the dataset. Future studies should incorporate hemodynamic data and standardized mechanical ventilation modes to further elucidate the relationship between Pmean trajectories and mortality. Finally, we examined only the impact of Pmean trajectory—a single indicator—on mortality and AKI outcomes. Future research should consider incorporating trends in other relevant indicators to improve predictive accuracy for disease outcomes.

## 5 Conclusion

The trajectory of Pmean within the first 72 h is significantly associated with mortality and AKI risk, highlighting the importance of early ventilatory management and its potential impact on patient outcomes. Relative to patients with low and stable Pmean levels, those with medium-ascend trajectories faced an increased risk of both mortality and AKI, while patients with high-stable or higher-stable Pmean trajectories were also at elevated risk for these adverse outcomes. Additional well-designed prospective studies are required to confirm and strengthen our findings.

## Data Availability

Publicly available datasets were analyzed in this study. This data can be found here: https://physionet.org/content/mimiciv/2.0/.

## Glossary

MIMIC-IV: Multiparameter intelligent monitoring in intensive care database IV
MV: Mechanical ventilation
ARDS: Acute respiratory distress syndrome
AKI: Acute kidney injury
GBTM: Group-based trajectory modeling
VILI: Ventilator-induced lung injury
TWA: Time-weighted averages
STROBE: Strengthening the reporting of observational studies in epidemiology
SQL: Structured query language
HR: Hazard ratio
CI: Confidence interval
ICU: Intensive care unit
IQR: Interquartile range
BMI: Body mass index
RRT: Renal replacement therapy
SOFA: Sequential organ failure assessment
SAPS II: Simplified acute physiology score II
CAD: Cerebrovascular disease
PEEP: Positive end-expiratory pressure
NMBA: Neuromuscular blocker agent
CMA: Causal mediation analysis
ACME: Average causal mediation effect
ADE: Average direct effect
