# Supplementary material for: Influence of the mean airway pressure trajectory on the mortality and AKI occurrence in septic shock patients with mechanical ventilation: insights from the MIMIC-IV database
Source: Front Med (Lausanne). 2025 Mar 5;12:1552336. doi: 10.3389/fmed.2025.1552336 (PMC11919853; doi:10.3389/fmed.2025.1552336)
Supplement: Supplementary file 1 [file Supplementary_file_1.docx]

| **Supplement Table 1.**  Percentage of missing data in the variables in the interest | | |  |
| --- | --- | --- | --- |
| Variable | Number of missing values | Proportion of missing values |  |
| Age(years) | 0 | 0 |  |
| Gender | 0 | 0 |  |
| BMI (kg/m2) | 142 | 14.8 |  |
| Ethnicity | 0 | 0 |  |
| SAPS II score | 0 | 0 |  |
| SOFA score | 0 | 0 |  |
| Malignancy | 0 | 0 |  |
| ARDS | 0 | 0 |  |
| CAD | 0 | 0 |  |
| CHF | 0 | 0 |  |
| COPD | 0 | 0 |  |
| Fluid balance, (L) | 0 | 0 |  |
| NMBA use, n (%) | 0 | 0 |  |
| RRT use | 0 | 0 |  |
| Heart rate, (bpm) | 0 | 0 |  |
| MAP, (mmHg) | 0 | 0 |  |
| Respiratory rate, (bpm) | 0 | 0 |  |
| SpO2, % | 0 | 0 |  |
| PF ratio | 9 | 0.9414 |  |
| Chloride, (mEq/L) | 1 | 0.1046 |  |
| Creatinine, (g/dL) | 1 | 0.1046 |  |
| Hemoglobin, (g/dl) | 1 | 0.1046 |  |
| Lactate (mmol/L) | 0 | 0 |  |
| PaCO2(mmHg) | 2 | 0.2092 |  |
| PH | 2 | 0.2092 |  |
| Platelet, (k/uL) | 1 | 0.1046 |  |
| Sodium, ( (mEq/L)) | 1 | 0.1046 |  |
| Neutrophil, (k/uL) | 212 | 22.1 |  |
| Bicarbonate, ( (mEq/L) | 1 | 0.1046 |  |
| Mechanical power | 0 | 0 |  |
| PEEP | 0 | 0 |  |
| Driven pressure | 44 | 4.6025 |  |
| Tidal volume(ml) | 0 | 0 |  |
| BMI, body mass index; SOFA: Sequential Organ Failure Assessment, SAPSII, Simpliﬁed acute physiology score II, bpm: beats per minute, MAP: mean arterial blood pressure, SpO2 pulse oximetry, RRT: renal replacement therapy, COPD: chronic obstructive pulmonary disease, CAD: Cerebrovascular disease, PaCO2 arterial carbon dioxide tension, PaO2: arterial oxygen tension, PEEP: positive end-expiratory pressure, NMBA: neuromuscular blocker agent | | |  |
|  |  |  |  |
|  |  |  |  |
|  |  |  |  |
|  |  |  |  |
|  |  |  |  |
|  |  |  |  |

| **Supplement Table 2.** Fit statistics for diﬀerent number of trajectory groups. | | | | | | | |
| --- | --- | --- | --- | --- | --- | --- | --- |
| Trajectory groups |  | Avepp(%) | Percent of patients with posterior probability < 70% (per trajectory group) | BIC | AIC | 2△BIC | Entropy |
| 2Group | 11 | 0.996/0.989 | 72.3/27.6 | -49415.27 | -49400.68 |  | 0.979 |
| 2Group | 22 | 0.996/0.990 | 72.2/ 27.7 | -49281.83 | -49262.38 | 266.88 | 0.979 |
| 3Group | 111 | 0.990/0.972/0.983 | 50.7/34.3/ 14.9 | -47399.46 | -47377.58 | 3764.74 | 0.957 |
| 3Group | 222 | 0.9870.979/0.998 | 51.2/ 34.8/13.8 | -47178.93 | -47149.75 | 4441.06 | 0.964 |
| 3Group | 333 | 0.987/0.979/0.998 | 51.2/34.8/13.8 | -47151.4 | -47114.93 | 1375.64 | 0.964 |
| 4Group | 1111 | 0.979/0.963/0.971/0.998 | 40.3/33.5/19.0/6.9 | -46491.1 | -46461.93 | 1306 | 0.951 |
| 4Group | 2222 | 0.975/0.960/0.978/0.990 | 41.7/32.8/18.4/6.9 | -46252.08 | -46213.18 | 239.02 | 0.948 |
| 5Group | 11111 | 0.971/0.956/0.947/0.973/0.997 | 36.6/30.1/17.2/12.5/3.3 | -46016.71 | -45980.23 | 465.9 | 0.942 |
| 5Group | 22222 | 0.948/0.978/0.944/0.981/0.981 | 22.7/40.1/13.5/16.8/6.7 | -45744.04 | -45695.41 | 272.67 | 0.947 |
| 5Group | 23133 | 0.985/0.925/0.952/0.985/0.982 | 39.4/13.5/23.2/17.1/6.8 | -45695.45 | -45641.96 | 97.18 | 0.944 |
| 5Group | 33333 | 0.987/0.931/0.951/0.977/0.992 | 39.9/15.1/21.6/16.7/6.5 | -45659.15 | -45598.37 | 87.18 | 0.947 |
| 6Group | 222222 | 0.983/0.975/0.963/0.937/0.972/0.991 | 7.8/37.6/17.8/23.9/8.9/3.8 | -45289.16 | -45230.81 | 735.12 | 0.943 |
| Avepp;Average post-test grouping probability;AIC: Akaike information criterion; BIC: Bayesian information criteria;2 ΔBIC: Twice the diﬀerence between the BIC values. | | | | | | | |

| **Supplement Table 3.** Group (23133) trajectory model parameter estimation | | | | | |
| --- | --- | --- | --- | --- | --- |
| Group | Parameter | Estimate | Standard Error | T | P |
| Group1 | Intercept | 9.42634 | 0.08457 | 111.466 | <0.001 |
|  | Linear | -0.04995 | 0.00562 | -8.883 | <0.001 |
|  | Quadratic | 0.00042 | 0.00008 | 5.283 | <0.001 |
| Group2 | Intercept | 13.35994 | 0.18843 | 70.902 | <0.001 |
|  | Linear | 0.17081 | 0.02191 | 7.796 | <0.001 |
|  | Quadratic | -0.0092 | 0.00079 | -11.667 | <0.001 |
|  | Cubic | 0.00008 | 0.00001 | 11.155 | <0.001 |
| Group3 | Intercept | 11.25071 | 0.1262 | 89.147 | <0.001 |
|  | Linear | 0.02822 | 0.003 | 9.404 | <0.001 |
| Group4 | Intercept | 14.44164 | 0.17334 | 83.314 | <0.001 |
|  | Linear | 0.22857 | 0.02018 | 11.327 | <0.001 |
|  | Quadratic | -0.00537 | 0.00068 | -7.924 | <0.001 |
|  | Cubic | 0.00003 | 0.00001 | 5.25 | <0.001 |
| Group5 | Intercept | 17.78255 | 0.24384 | 72.926 | <0.001 |
|  | Linear | 0.37626 | 0.03135 | 12.002 | <0.001 |
|  | Quadratic | -0.00893 | 0.00104 | -8.627 | <0.001 |
|  | Cubic | 0.00006 | 0.00001 | 6.298 | <0.001 |

| **Supplement Table 4.** Association between Pmean levels at different time points within 72hours after ICU admission and 30-day mortality | | | | | | | | | | | | | | | | | |  |  |  |  |
| --- | --- | --- | --- | --- | --- | --- | --- | --- | --- | --- | --- | --- | --- | --- | --- | --- | --- | --- | --- | --- | --- |
| Pmean levels at different time points | | | | | | n.total | | Univariable model | | | | | Multivariable model | | | | |  |  |  |  |
|  |  |  |  |  |  |  |  | HR_95%CI | | | P_value | | HR_95%CI | | | P_value | |  |  |  |  |
| TWA-Pmean^a^, (cm H_2_O) | | | | | | 956 | | 1.02 (1~1.05) | | | 0.111 | | 1 (0.97~1.04) | | | 0.923 | |  |  |  |  |
| TWA-Pmean^b^,(cm H2O) | | | | | | 956 | | 1.04 (1.01~1.06) | | | 0.002 | | 1.05 (1.01~1.08) | | | 0.004 | |  |  |  |  |
| TWA-Pmean^c^,(cm H2O) | | | | | | 956 | | 1.06 (1.03~1.08) | | | <0.001 | | 1.09 (1.06~1.12) | | | <0.001 | |  |  |  |  |
| ICU: intensive care unit; HR: hazard ratio; CI: confidence interval. Multivariable model adjusted forage, gender, BMI, SAPS II score, SOFA score, fluid balance, respiratory rate, SpO2, PF ratio,PH,bicarbonate,lactate and sodium; ^a^  First 24-hour;^b^ Second 24-hour; ^c^ Third 24-hour | | | | | | | | | | | | | | | | | |  |  |  |  |
| **Supplement Table 5.** The mediation effect of Cumulative fluid balance on the association between Pmean trajectory and mortality or AKI occurence | | | | | | | | | | | | | | | | | | | |  |  |
| Model Pathways | | **Mediation effect (95% CI), P value** | | | | | | | | | | | | | | | | | |  |  |
|  |  | Total effect | | | | Mediation effect | | | | | Direct effect | | | | | Proportion of mediation | | | |  |  |
| 30-day mortality | | 0.08 | (0.027,0.139) | <0.001 | | -0.005 | | (-0.014,0.005) | 0.296 | | 0.085 | | (0.031,0.142) | <0.001 | | -6.30% | | (-0.286,0.072) | 0.296 |  |  |
| ICU mortality | | 0.108 | (0.063,0.155) | <0.001 | | -0.002 | | ( -0.010, 0.006) | 0.67 | | 0.11 | | (0.063,0.157) | <0.001 | | -1.80% | | (-0.110,0.064) | 0.67 |  |  |
| Hospital mortality | | 0.091 | (0.042,0.145) | <0.001 | | -0.002 | | (-0.010,0.007) | 0.726 | | 0.093 | | 0.043,0.147) | <0.001 | | -2.10% | | (-0.146,0.104) | 0.726 |  |  |
| AKI occurrence | | 0.083 | (0.043, 0.140) | <0.001 | | 0.017 | | (0.009, 0.029) | <0.001 | | 0.066 | | (0.027, 0.115) | <0.001 | | 20.50% | | (0.106,0.40) | <0.001 |  |  |
|  | | | | | | | | | | | | | | | | | | | |  |  |
|  |  |  |  |  |  |  |  |  |  |  |  |  |  |  |  |  |  |  |  |  |  |
|  |  |  |  |  |  |  |  |  |  |  |  |  |  |  |  |  |  |  |  |  |  |
|  |  |  |  |  |  |  |  |  |  |  |  |  |  |  |  |  |  |  |  |  |  |
| \| **Supplement Table 6.** Subgroup analysis for the association of Pmean levels trajectories with risk of 30-day mortality. \| \| \| \| \| \| \| \| \| \| --- \| --- \| --- \| --- \| --- \| --- \| --- \| --- \| --- \| \| Subgroup \| Variable \| HR_95%CI \| P_value \| Subgroup \| Variable \| HR_95%CI \| P_value \| P for.interaction \| \| Age, (years) \|  \|  \|  \| Age, (years) \|  \|  \|  \| 0.786 \| \| <60 \| Group1 \| reference \|  \| ≥60 \| Group1 \| reference \|  \|  \| \|  \| Group2 \| 0.79 (0.4~1.57) \| 0.507 \|  \| Group2 \| 0.89 (0.54~1.47) \| 0.655 \|  \| \|  \| Group3 \| 1.26 (0.76~2.09) \| 0.375 \|  \| Group3 \| 1.51 (1.02~2.24) \| 0.037 \|  \| \|  \| Group4 \| 1.98 (1.08~3.63) \| 0.028 \|  \| Group4 \| 1.29 (0.79~2.12) \| 0.306 \|  \| \|  \| Group5 \| 2.98 (1.43~6.22) \| 0.004 \|  \| Group5 \| 2.11 (1~4.47) \| 0.051 \|  \| \| Female \|  \|  \|  \| Male \|  \|  \|  \| 0.546 \| \|  \| Group1 \| reference \|  \|  \| Group1 \| reference \|  \|  \| \|  \| Group2 \| 1.05 (0.59~1.84) \| 0.877 \|  \| Group2 \| 0.75 (0.43~1.31) \| 0.316 \|  \| \|  \| Group3 \| 1.52 (0.95~2.42) \| 0.079 \|  \| Group3 \| 1.47 (0.99~2.21) \| 0.059 \|  \| \|  \| Group4 \| 1.91 (1.09~3.37) \| 0.025 \|  \| Group4 \| 1.23 (0.73~2.06) \| 0.433 \|  \| \|  \| Group5 \| 2.73 (1.29~5.79) \| 0.009 \|  \| Group5 \| 2.62 (1.29~5.34) \| 0.008 \|  \| \| BMI (kg/m2) \|  \|  \|  \| BMI (kg/m2) \|  \|  \|  \| 0.522 \| \| ＜30 \| Group1 \| reference \|  \| ≥30 \| Group1 \| reference \|  \|  \| \|  \| Group2 \| 0.78 (0.48~1.26) \| 0.302 \|  \| Group2 \| 0.95 (0.48~1.91) \| 0.891 \|  \| \|  \| Group3 \| 1.31 (0.9~1.89) \| 0.155 \|  \| Group3 \| 1.55 (0.89~2.71) \| 0.124 \|  \| \|  \| Group4 \| 1.42 (0.88~2.31) \| 0.152 \|  \| Group4 \| 1.51 (0.82~2.8) \| 0.19 \|  \| \|  \| Group5 \| 3.65 (1.78~7.49) \| 0.143 \|  \| Group5 \| 2.22 (1.03~4.82) \| 0.043 \|  \| \| Prone position ventilation \|  \|  \|  \| Prone position ventilation \|  \|  \|  \| 0.708 \| \| No \| Group1 \| reference \|  \| Yes \| Group1 \| reference \|  \|  \| \|  \| Group2 \| 0.87 (0.58~1.3) \| 0.493 \|  \| Group2 \| 2.59 (0.37~18.27) \| 0.339 \|  \| \|  \| Group3 \| 1.47 (1.08~2.01) \| 0.015 \|  \| Group3 \| 1.39 (0.26~7.48) \| 0.703 \|  \| \|  \| Group4 \| 1.34 (0.92~1.96) \| 0.127 \|  \| Group4 \| 12.37 (0.88~174.12) \| 0.062 \|  \| \|  \| Group5 \| 2.26 (1.36~3.75) \| 0.002 \|  \| Group5 \| 20.78 (1.1~392.29) \| 0.043 \|  \| \| NMBA use \|  \|  \|  \| NMBA use \|  \|  \|  \| 0.494 \| \| No \| Group1 \| reference \|  \| Yes \| Group1 \| reference \|  \|  \| \|  \| Group2 \| 0.95 (0.64~1.4) \| 0.781 \|  \| Group2 \| 0.2 (0.01~2.82) \| 0.235 \|  \| \|  \| Group3 \| 1.46 (1.06~2) \| 0.021 \|  \| Group3 \| 1.04 (0.2~5.43) \| 0.966 \|  \| \|  \| Group4 \| 1.46 (0.98~2.16) \| 0.062 \|  \| Group4 \| 4.93 (0.69~35.22) \| 0.111 \|  \| \|  \| Group5 \| 2.56 (1.49~4.4) \| 0.001 \|  \| Group5 \| 2.05 (0.24~17.15) \| 0.509 \|  \| \| SAPS II score \|  \|  \|  \| SAPS II score \|  \|  \|  \| 0.676 \| \| <49 \| Group1 \| reference \|  \| ≥49 \| Group1 \| reference \|  \|  \| \|  \| Group2 \| 0.96 (0.6~1.55) \| 0.868 \|  \| Group2 \| 0.82 (0.41~1.63) \| 0.571 \|  \| \|  \| Group3 \| 1.43 (0.98~2.09) \| 0.06 \|  \| Group3 \| 1.5 (0.88~2.58) \| 0.138 \|  \| \|  \| Group4 \| 1.45 (0.89~2.38) \| 0.14 \|  \| Group4 \| 1.39 (0.76~2.56) \| 0.288 \|  \| \|  \| Group5 \| 2.28 (1.17~4.47) \| 0.016 \|  \| Group5 \| 2.58 (1.18~5.66) \| 0.018 \|  \| \| SOFA score \|  \|  \|  \| SOFA score \|  \|  \|  \| 0.67 \| \| <10 \| Group1 \| reference \|  \| >10 \| Group1 \| reference \|  \|  \| \|  \| Group2 \| 1.11 (0.45~2.73) \| 0.822 \|  \| Group2 \| 0.84 (0.54~1.3) \| 0.429 \|  \| \|  \| Group3 \| 1.93 (1.07~3.48) \| 0.03 \|  \| Group3 \| 1.28 (0.9~1.82) \| 0.176 \|  \| \|  \| Group4 \| 1.61 (0.56~4.63) \| 0.373 \|  \| Group4 \| 1.42 (0.94~2.13) \| 0.095 \|  \| \|  \| Group5 \| 1.03 (0.12~9.05) \| 0.98 \|  \| Group5 \| 2.62 (1.52~4.5) \| 0.03 \|  \| \| ARDS \|  \|  \|  \| ARDS \|  \|  \|  \| 0.535 \| \| No \| Group1 \| reference \|  \| Yes \| Group1 \| reference \|  \|  \| \|  \| Group2 \| 0.81 (0.52~1.26) \| 0.349 \|  \| Group2 \| 1.27 (0.51~3.18) \| 0.605 \|  \| \|  \| Group3 \| 1.36 (0.97~1.91) \| 0.075 \|  \| Group3 \| 1.97 (0.95~4.09) \| 0.069 \|  \| \|  \| Group4 \| 1.27 (0.8~1.99) \| 0.311 \|  \| Group4 \| 2.79 (1.27~6.12) \| 0.01 \|  \| \|  \| Group5 \| 2.51 (1.36~4.61) \| 0.003 \|  \| Group5 \| 4.14 (1.49~11.48) \| 0.006 \|  \| \| CHF \|  \|  \|  \| CHF \|  \|  \|  \| 0.461 \| \| No \| Group1 \| reference \|  \| Yes \| Group1 \| reference \|  \|  \| \|  \| Group2 \| 0.92 (0.59~1.42) \| 0.693 \|  \| Group2 \| 0.94 (0.39~2.26) \| 0.884 \|  \| \|  \| Group3 \| 1.22 (0.87~1.71) \| 0.257 \|  \| Group3 \| 2.4 (1.21~4.77) \| 0.012 \|  \| \|  \| Group4 \| 1.29 (0.84~1.97) \| 0.245 \|  \| Group4 \| 2.42 (1.1~5.33) \| 0.029 \|  \| \|  \| Group5 \| 2.11 (1.2~3.72) \| 0.01 \|  \| Group5 \| 6.7 (2.03~22.08) \| 0.002 \|  \| \| OR,odds ratio; CI, confidence interval; BMI: body mass index;SAPSII, Simpliﬁed acute physiology score II, SOFA, Sequential Organ Failure Assessment;NMBA,neuromuscular blocker agent; CHF, congestive heart failure; ;ARDS, acute respiratory distress syndrome \| \| \| \| \| \| \| \| \|  \| **Supplement Table 7:**  Hazard ratio or odds ratio for risks of mortality by different Pmean trajectory patterns after 24 hours of mechanical ventilation. \| \| \| \| \| \| \| \|  \|  \| \| --- \| --- \| --- \| --- \| --- \| --- \| --- \| --- \| --- \| --- \| \| Variable \| n.total \| Crude \|  \| Model1 \|  \| Model2 \|  \| Model3 \|  \| \| HR/OR (95% CI) \| P_value \| HR/OR (95% CI) \| P_value \| HR/OR (95% CI) \| P_value \| HR/OR (95% CI) \| P_value \| \| 30-day mortality \|  \|  \|  \|  \|  \|  \|  \|  \|  \| \| Group1 \| 408 \| reference \|  \| reference \|  \| reference \|  \| reference \|  \| \| Group2 \| 150 \| 0.86 (0.6~1.23) \| 0.41 \| 0.87 (0.61~1.25) \| 0.446 \| 0.83 (0.56~1.22) \| 0.331 \| 0.86 (0.6~1.23) \| 0.41 \| \| Group3 \| 205 \| 1.57 (1.19~2.06) \| 0.001 \| 1.78 (1.35~2.36) \| <0.001 \| 1.73 (1.28~2.33) \| <0.001 \| 1.57 (1.19~2.06) \| 0.001 \| \| Group4 \| 137 \| 1.59 (1.17~2.16) \| 0.003 \| 1.87 (1.36~2.58) \| <0.001 \| 1.88 (1.29~2.73) \| 0.001 \| 1.59 (1.17~2.16) \| 0.003 \| \| Group5 \| 56 \| 2.38 (1.6~3.52) \| <0.001 \| 3.32 (2.19~5.03) \| <0.001 \| 3.17 (1.91~5.24) \| <0.001 \| 2.38 (1.6~3.52) \| <0.001 \| \| ICU mortality \|  \|  \|  \|  \|  \|  \|  \|  \|  \| \| Group1 \| 408 \| reference \|  \| reference \|  \| reference \|  \| reference \|  \| \| Group2 \| 150 \| 1 (0.62~1.6) \| 0.994 \| 1.02 (0.63~1.65) \| 0.938 \| 0.98 (0.58~1.66) \| 0.945 \| 0.87 (0.51~1.5) \| 0.621 \| \| Group3 \| 205 \| 2.45 (1.69~3.57) \| <0.001 \| 2.89 (1.96~4.28) \| <0.001 \| 2.93 (1.92~4.47) \| <0.001 \| 2.9 (1.88~4.48) \| <0.001 \| \| Group4 \| 137 \| 2.47 (1.62~3.78) \| <0.001 \| 3.07 (1.95~4.81) \| <0.001 \| 3.2 (1.87~5.48) \| <0.001 \| 3.22 (1.84~5.62) \| <0.001 \| \| Group5 \| 56 \| 4.47 (2.51~7.98) \| <0.001 \| 6.69 (3.58~12.5) \| <0.001 \| 6.97 (3.27~14.85) \| <0.001 \| 7.5 (3.46~16.26) \| <0.001 \| \| hospital mortality \|  \|  \|  \|  \|  \|  \|  \|  \|  \| \| Group1 \| 408 \| reference \|  \| reference \|  \| reference \|  \| reference \|  \| \| Group2 \| 150 \| 0.83 (0.54~1.29) \| 0.413 \| 0.83 (0.53~1.29) \| 0.407 \| 0.75 (0.46~1.23) \| 0.254 \| 0.68 (0.41~1.12) \| 0.127 \| \| Group3 \| 205 \| 1.83 (1.29~2.61) \| 0.001 \| 2.22 (1.53~3.21) \| <0.001 \| 2.14 (1.43~3.2) \| <0.001 \| 2.1 (1.4~3.17) \| <0.001 \| \| Group4 \| 137 \| 1.88 (1.26~2.82) \| 0.002 \| 2.35 (1.52~3.61) \| <0.001 \| 2.32 (1.38~3.88) \| 0.001 \| 2.32 (1.37~3.94) \| 0.002 \| \| Group5 \| 56 \| 3.05 (1.73~5.38) \| <0.001 \| 4.8 (2.59~8.87) \| <0.001 \| 4.57 (2.19~9.55) \| <0.001 \| 4.72 (2.23~9.96) \| <0.001 \| \| Model 1: adjusted for age, gender, and BMI.  Model 2: adjusted for age, gender, BMI, SAPS II score, SOFA score, fluid balance respiratory rate, SpO_2_ and PF ratio.  Model 3: adjusted for age, gender, BMI, SAPS II score, SOFA score, fluid balance, respiratory rate, SpO_2_, PF ratio, PH, Bicarbonate, Lactate and sodium \| \| \| \| \| \| \| \| \| \| \| \| \|   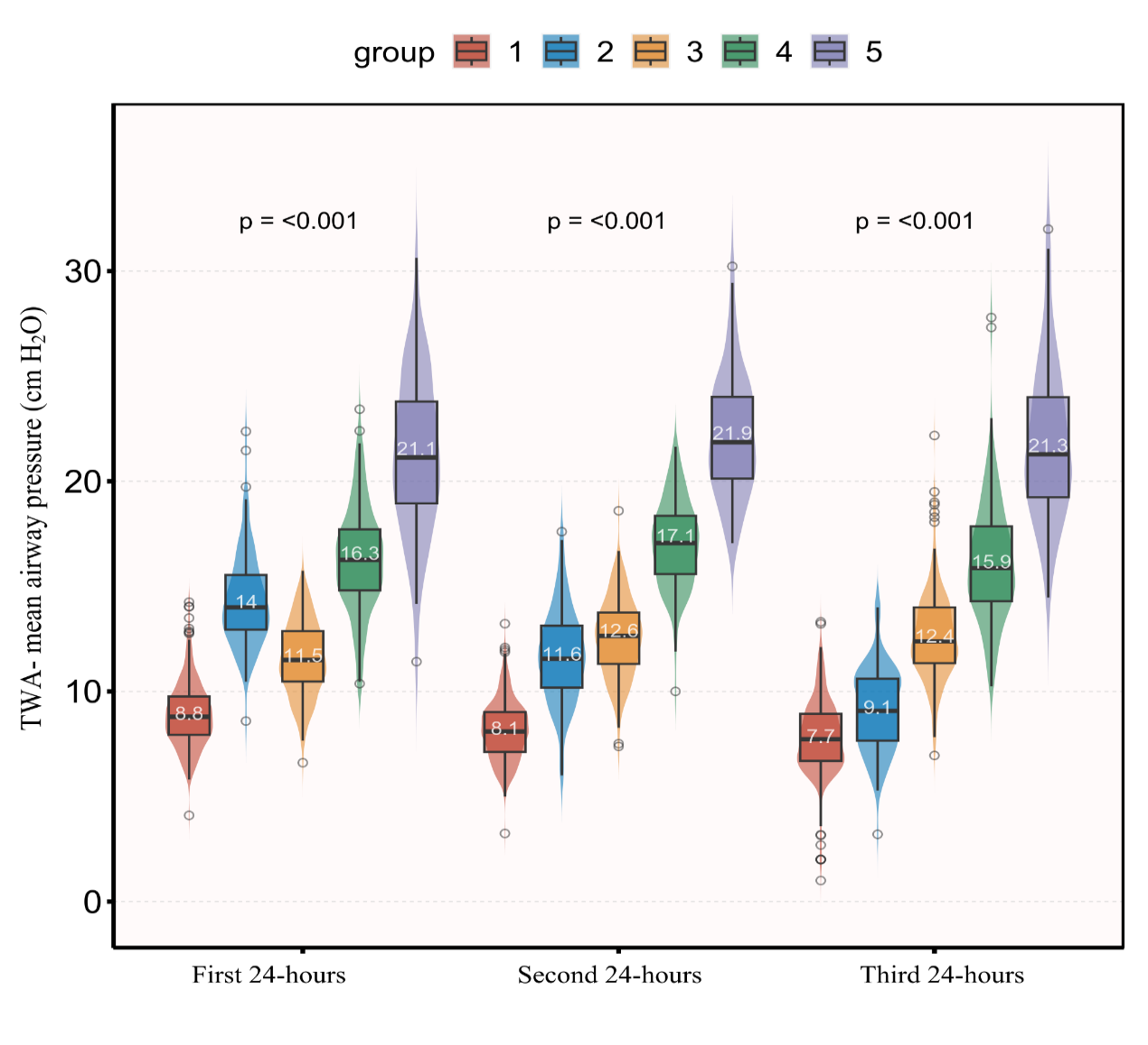  **Supplement Figure 1**. Daily mean airway pressure in the five subgroups with different trajectory patterns.  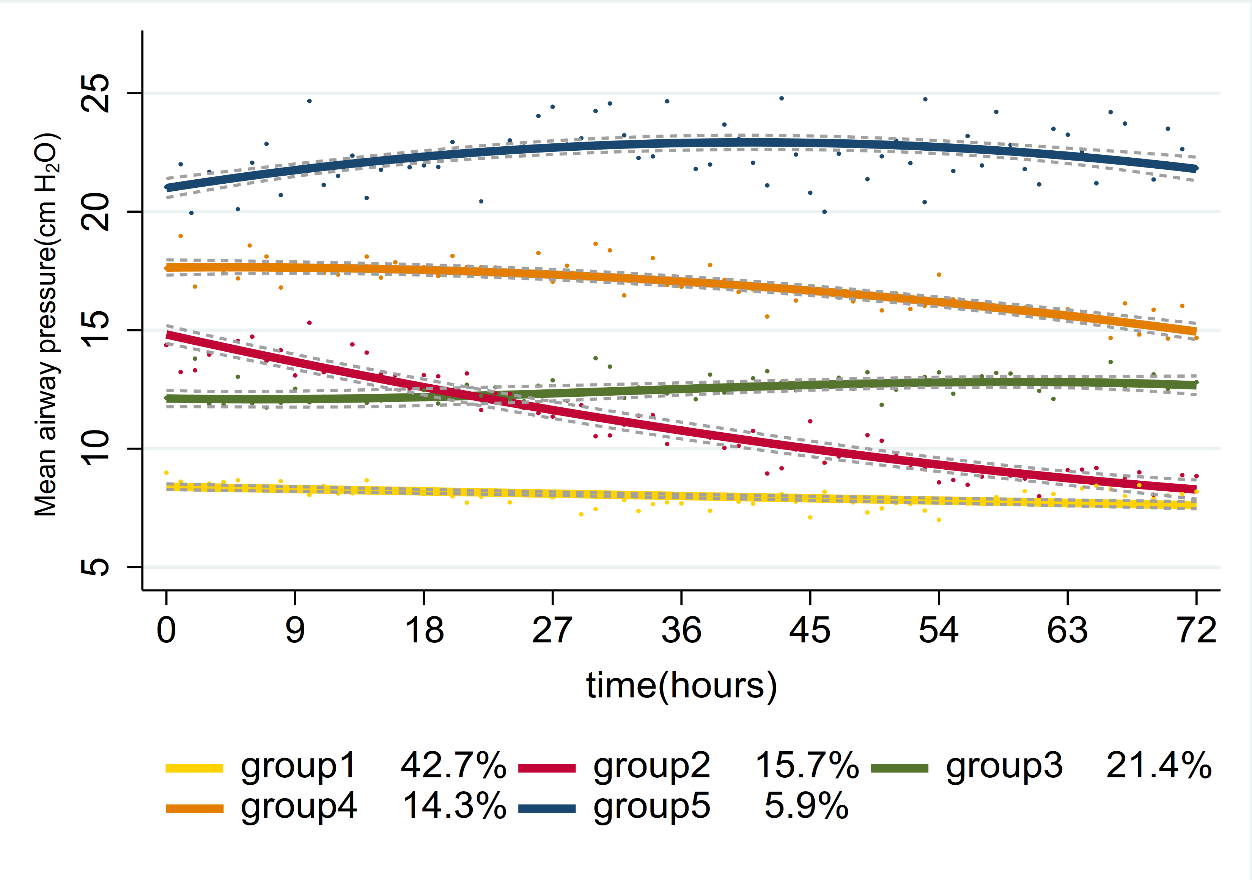  **Supplement Figure 2**. Mean airway pressure-based trajectories of patients with septic shock with mechanical ventilation after 24 hours of mechanical ventilation.  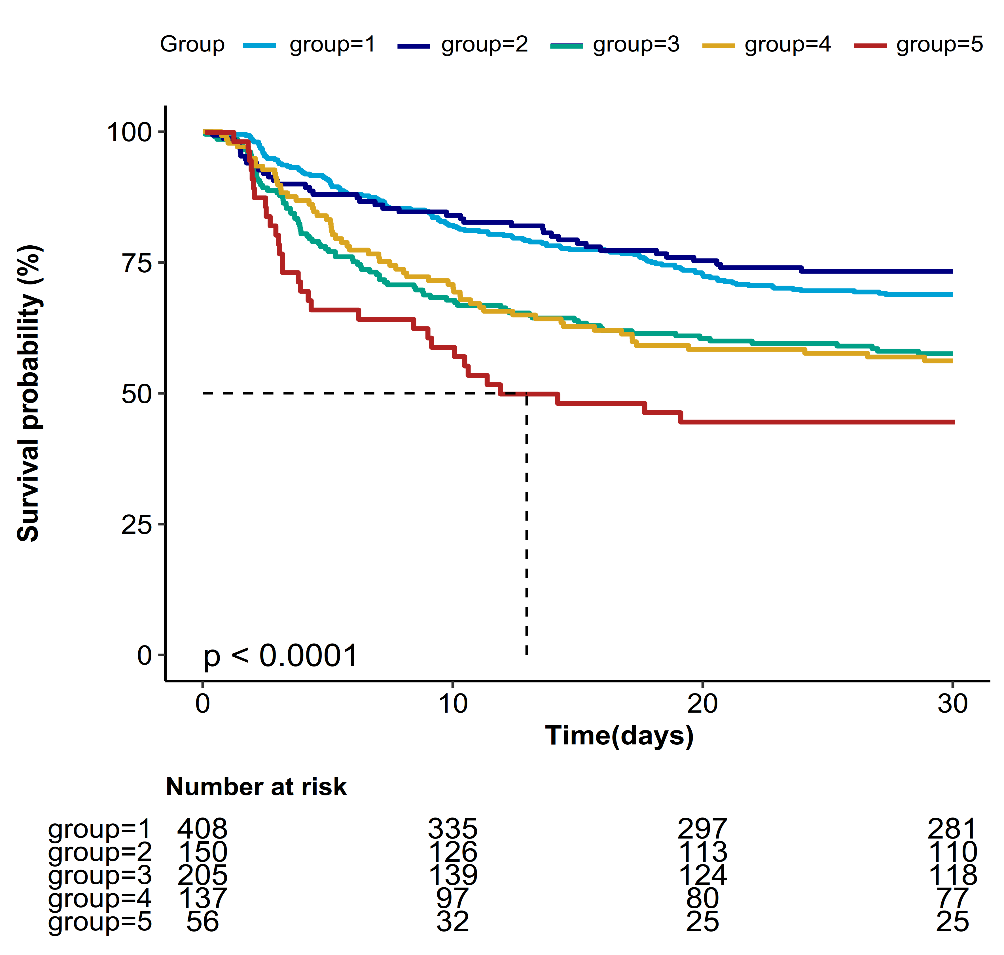  **Supplement Figure 3.** Kaplan–Meier survival estimates of 30-day mortality among each mean airway pressure trajectory after 24 hours of mechanical ventilation  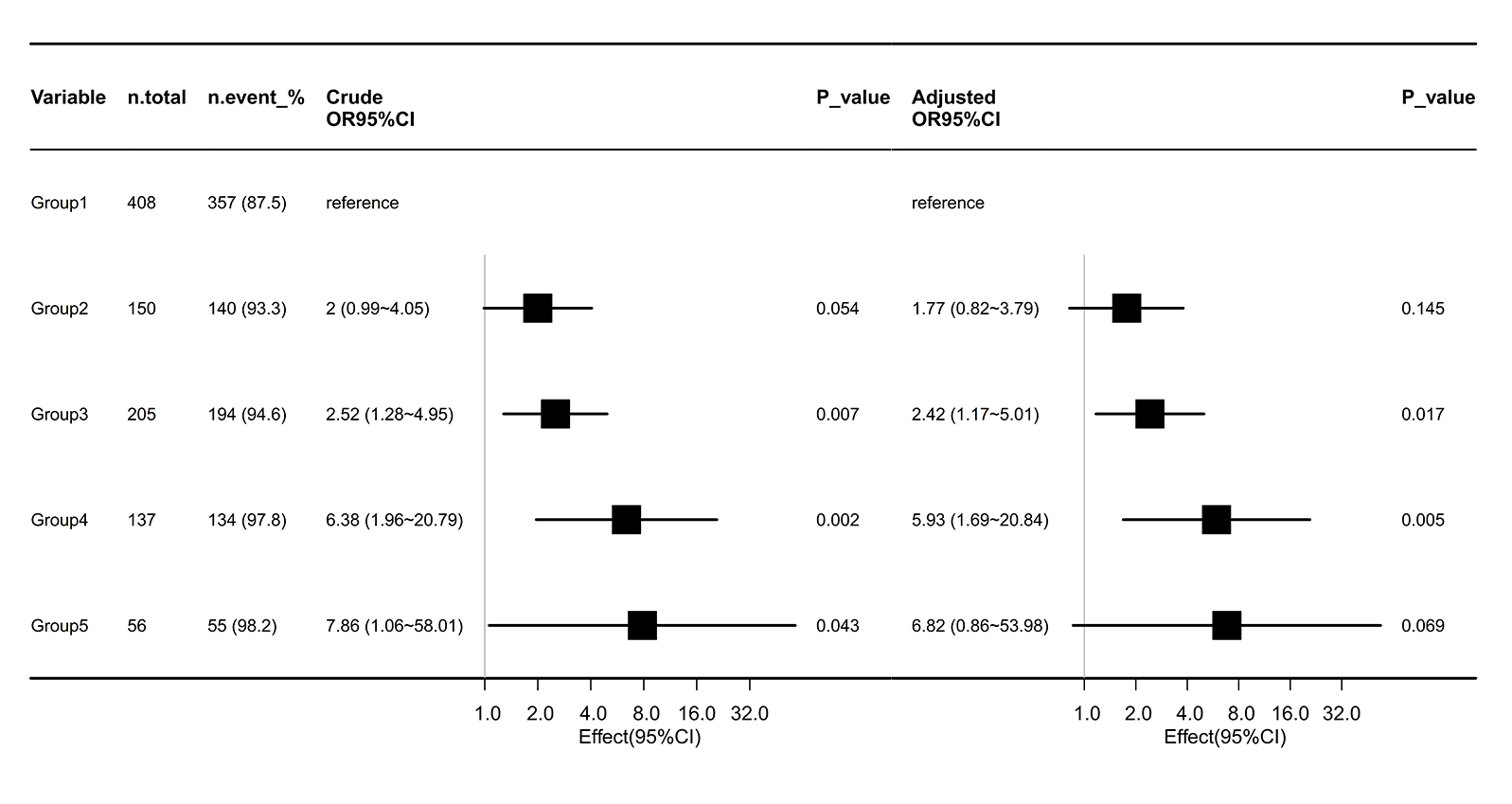  **Supplement Figure 4.** Association between mean airway pressure trajectory and AKI occurrence after 24 hours of mechanical ventilation. .OR: Odds Ratio; CI, confidence interval  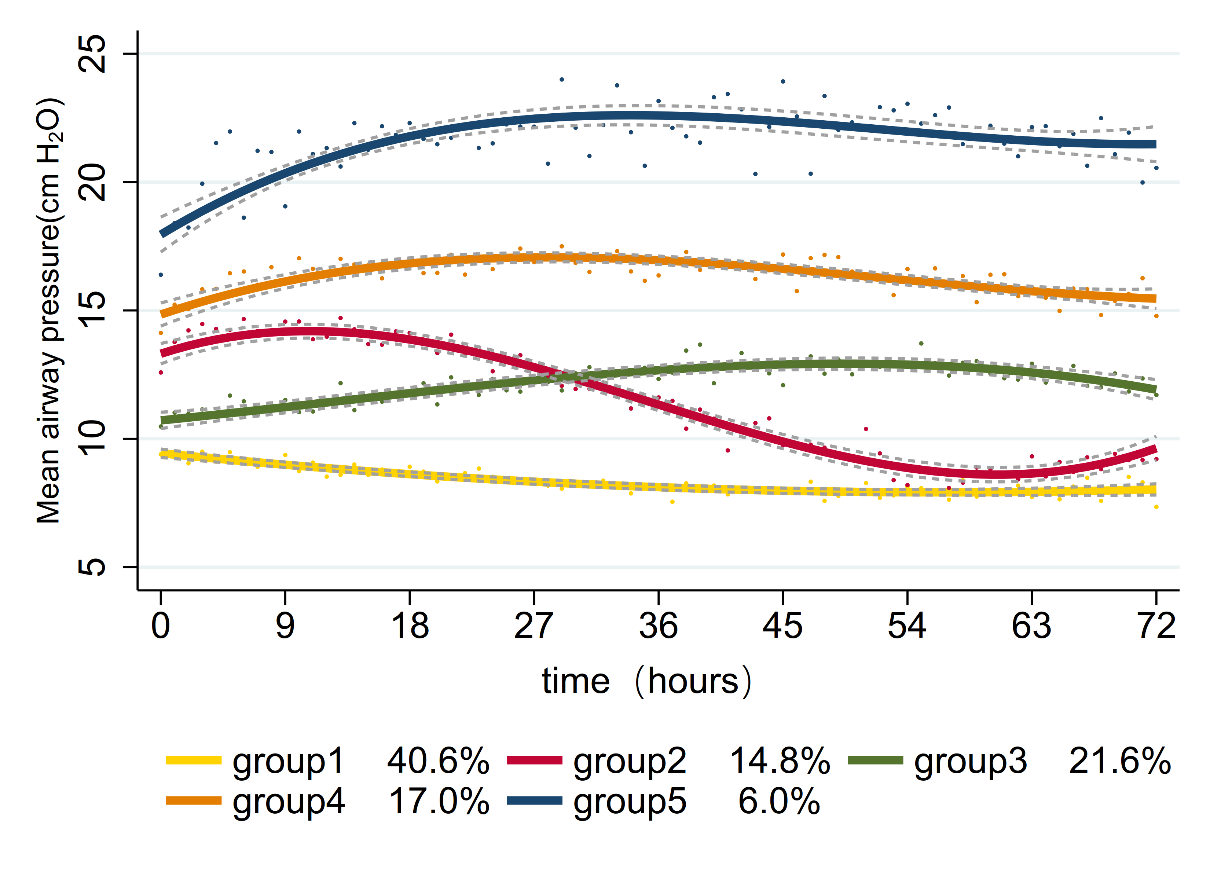  **Supplement Figure 5.** Mean airway pressure trajectories in mechanically ventilated patients with septic shock after the exclusion of RRT patients  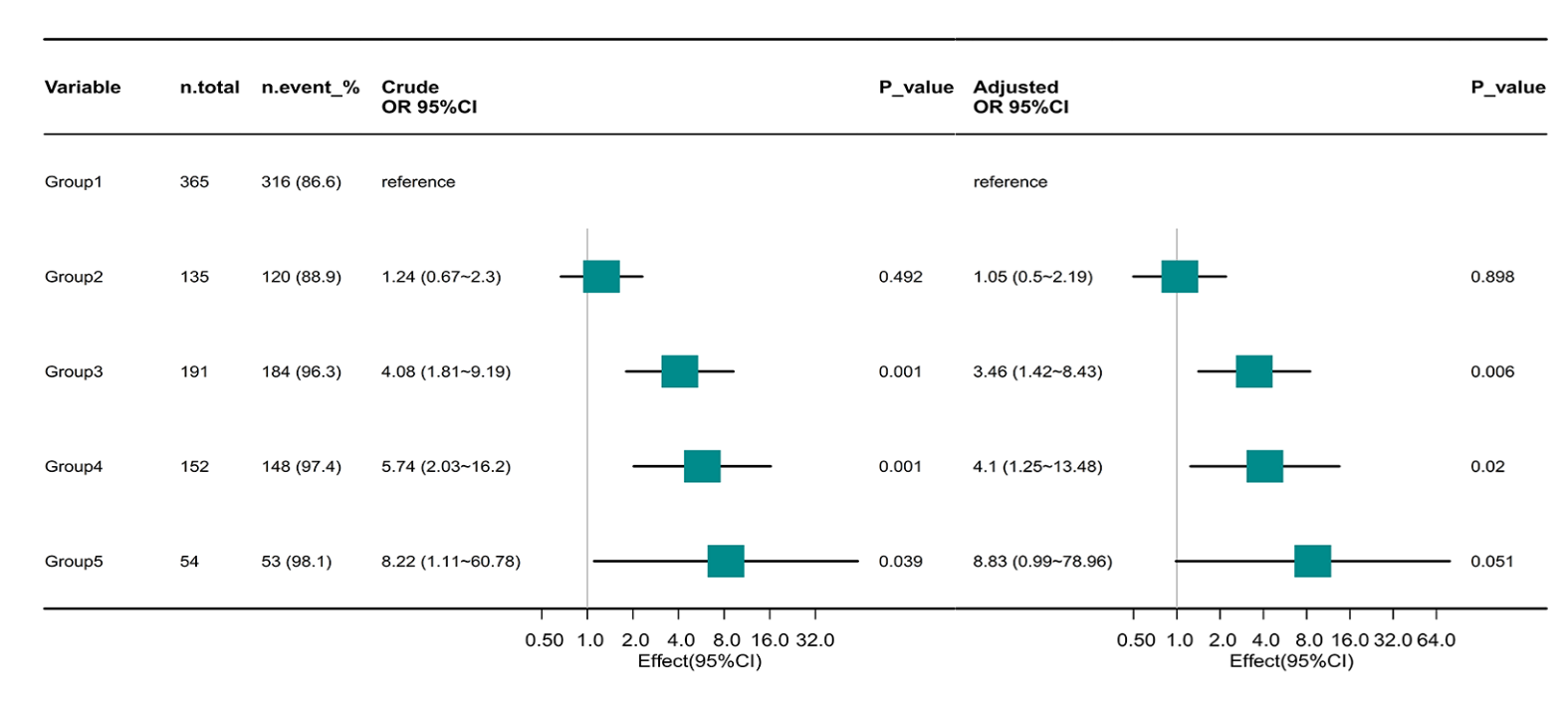 | | | | | | | | | | | | | | | | | | | | |  |
|  |  |  |  |  |  |  |  |  |  |  |  |  |  |  |  |  |  |  |  |  |  |

**Supplement Figure 6.** Association between mean airway pressure trajectory and AKI occurrence after after excluding patients with RRT.OR: Odds Ratio; CI, confidence interval.
